# Bioprospecting for the soil-derived actinobacteria and bioactive secondary metabolites on the Western Qinghai-Tibet Plateau

**DOI:** 10.3389/fmicb.2023.1247001

**Published:** 2023-10-11

**Authors:** Lifang Liu, Yuyu Liu, Shaowei Liu, Arina A. Nikandrova, Arina N. Imamutdinova, Dmitrii A. Lukianov, Ilya A. Osterman, Petr V. Sergiev, Benyin Zhang, Dejun Zhang, Feina Li, Chenghang Sun

**Affiliations:** ^1^Department of Microbial Chemistry, Institute of Medicinal Biotechnology, Chinese Academy of Medical Sciences and Peking Union Medical College, Beijing, China; ^2^Beijing Key Laboratory of Antimicrobial Agents, Institute of Medicinal Biotechnology, Chinese Academy of Medical Sciences and Peking Union Medical College, Beijing, China; ^3^Center of Life Sciences, Skolkovo Institute of Science and Technology, Moscow, Russia; ^4^Department of Biology, Lomonosov Moscow State University, Moscow, Russia; ^5^Department of Chemistry, Lomonosov Moscow State University, Moscow, Russia; ^6^College of Eco-Environmental Engineering, Qinghai University, Xining, China; ^7^Laboratory of Respiratory Diseases, Beijing Key Laboratory of Pediatric Respiratory Infection Diseases, Beijing Pediatric Research Institute, Beijing Children’s Hospital, Capital Medical University, Beijing, China; ^8^Key Laboratory of Major Diseases in Children, Ministry of Education, National Clinical Research Center for Respiratory Diseases, National Center for Children’s Health, Beijing, China; ^9^State Key Laboratory of Plateau Ecology and Agriculture, Qinghai University, Xining, China

**Keywords:** Qinghai-Tibet Plateau, actinobacteria, *Amycolatopsis*, rifamycins, GNPs

## Abstract

**Introduction:**

The increase in incidence of multidrug-resistant bacteria and the inadequacy of new antimicrobial drugs have led to a widespread outbreak of bacterial antimicrobial resistance. To discover new antibiotics, biodiversity, and novelty of culturable actinobacteria dwelled in soil of the Western Qinghai-Tibet Plateau were investigated. By integrating antibacterial assay with omics tools, *Amycolatopsis* sp. A133, a rare actinobacterial strain and its secondary metabolites were further studied.

**Method:**

Culture-dependent method was used to obtain actinobacterial strains from two soil samples collected from Ali region in Qinghai-Tibet Plateau. The cultural extractions of representative strains were assayed against “ESKAPE” pathogens by paper-disk diffusion method and the double fluorescent protein reporter “pDualrep2” system. An *Amycolatopsis* strain coded as A133 was prioritized and its secondary metabolites were further analyzed and annotated by omics tools including antiSMASH and GNPS (Global Natural Social Molecular Networking). The predicted rifamycin analogs produced by *Amycolatopsis* sp. A133 were isolated and identified by chromatographic separation, such as Sephadex LH-20 and HPLC, and spectral analysis, such as NMR and UPLC-HRESI-MS/MS, respectively.

**Results:**

A total of 406 actinobacteria strains affiliated to 36 genera in 17 families of 9 orders were isolated. Out of 152 representative strains, 63 isolates exhibited antagonistic activity against at least one of the tested pathogens. Among them, 7 positive strains were identified by the “pDualrep2” system as either an inhibitor of protein translation or DNA biosynthesis. The cultural broth of *Amycolatopsis* sp. A133 exhibited a broader antimicrobial activity and can induce expression of TurboRFP. The secondary metabolites produced by strain A133 was annotated as rifamycins and zampanolides by antiSMASH and GNPS analysis. Five members of rifamycins, including rifamycin W, protorifamycin I, rifamycin W-M1, proansamycin B, and rifamycin S, were purified and identified. Rifamycin W-M1, was found as a new member of the naturally occurring rifamycin group of antibiotics.

**Discussion:**

Assisted by omics tools, the successful and highly efficient discovery of rifamycins, a group of clinically used antibiotics from actinobacteria in Ali area encouraged us to devote more energy to explore new antibiotics from the soils on the Western Tibetan Plateau.

## 1. Introduction

Nowadays, the increase in incidence of multidrug-resistant bacteria and the inadequacy of new antimicrobial drugs have led to a widespread outbreak of bacterial antimicrobial resistance (AMR; Huemer et al., 2020; Walesch et al., 2023). The emergence of AMR has evolved into one of the most significant threats to present-day medicine, necessitating the urgent need for new antimicrobials. Actinobacteria, renowned for its extensive production of natural products, have contributed to the production of approximately two-thirds of all known antibiotics of microbial origin, with about approximately half of all clinically used antibiotics currently derived from the actinobacterial source (Bibb, 2013). Nonetheless, the rediscovery of already-known antibiotics from actinobacteria, which dwell in conventional and easy-to-reach environmental niches, has decreased the efficacy of new antibiotic discovery aimed at curbing antimicrobial resistance.

To date, at least three strategies have been employed to breakthrough the bottleneck of rediscovering antibiotics. The first strategy involved in exploring actinobacteria from hard-to-reach and uncharted ecosystems, such as hot springs (Mahajan and Balachandran, 2017), caves (Rangseekaew and Pathom-Aree, 2019), deserts (Sayed et al., 2020), Northwestern Himalayas (Hussain et al., 2017; Shah et al., 2017), etc. The second strategy is similar with the first strategy, but focused more on screening rare actinobacteria and their hitherto underrepresented genera from unfamiliar settings, including *Nocardiopsis*, *Saccharothrix*, *Streptosporangium*, *Amycolatopsis*, *Lentzea,* etc. (Hussain et al., 2017; Ezeobiora et al., 2022). Third, utilizing omics technologies to predict and decipher potentially new antibiotics from complex bioactive mixtures. Our research group has successfully integrated each of these antibiotic discovery strategies with bioassay techniques to rapidly identify several new antibiotics derived from both mangroves and deserts (Lu et al., 2019; Wang et al., 2021). These encouraging results have inspired us to expand our search for pharmaceutical actinobacteria on the Western Qinghai-Tibet Plateau.

As a unique geographical unit, the Qinghai-Tibet Plateau (QTP) is encompassed by the Himalayas mountains in the southwest and the Kunlun and Aljin mountains in the northeast. This elevated plateau towers over southwestern China with an average elevation of 4,000 m, earning it “the roof of the world” or “the third pole in the earth-high pole” (Wu, 2001; Qiu, 2008). With an average altitude of 4,000 m above sea level, the QTP is significantly colder than any other region outside of the polar regions (Qiu, 2008). Among various parts of the plateau, the Western Qinghai-Tibet Plateau is especially known for its coldness, dryness, low oxygen levels, low cloud cover, and high radiation, making it an exceedingly secluded and inhospitable area (Ge et al., 2021). Consequently, these daunting physiological challenges and supply deficiencies have caused the Western Qinghai-Tibet Plateau as a hard-to-reach and sparsely populated area. Thus, this typical alpine ecosystem provides a quite pristine, natural laboratory for researchers.

**Figure 1:**
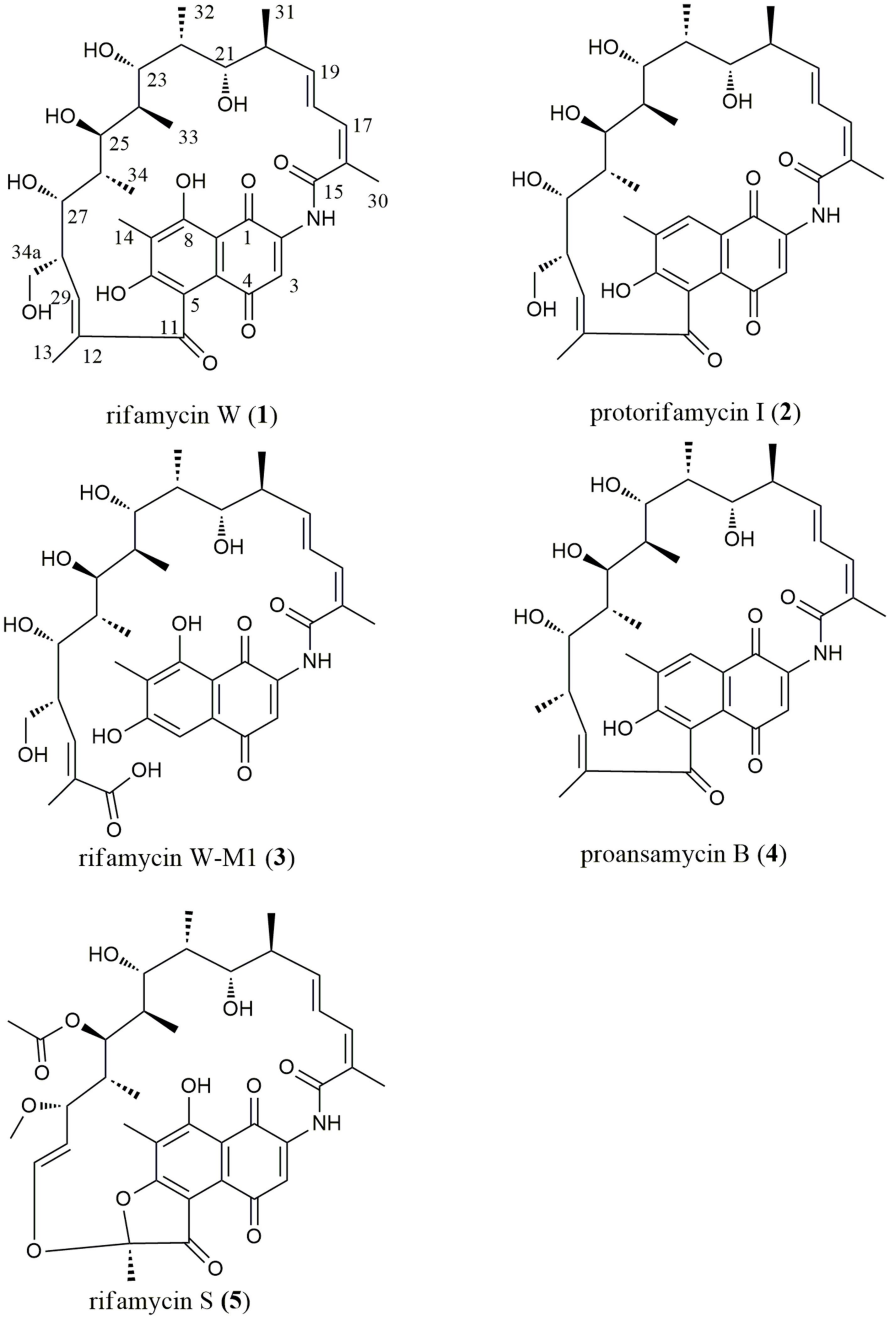
Chemical structures of five target compounds isolated from *Amycolatopsis* sp. A133.

This paper reported on the biodiversity and antimicrobial activity of actinobacteria isolated from soils on the Western Qinghai-Tibet Plateau. Additionally, antibacterial mechanisms of those bioactive strains were further studied using the high-throughput screening system “pDualrep2.” As a result, a rare actinobacterium, *Amycolatopsis* sp. A133 (strain A133) was prioritized to conduct annotation and prediction of bioactive secondary metabolites, by omics tools analysis such as antiSMASH and UPLC-QTOF-MS/MS based molecular networking. Through isolation and structural elucidation of the target compounds produced by strain A133, four known members, rifamycin W (**1**), protorifamycin I (**2**), proansamycin B (**4**), rifamycin S (**5**), and one new naturally occurring member of rifamycins, rifamycin W-M1 (**3**) were isolated and identified (Figure 1). The results demonstrated that the earlier decipherment assisted by omics tools can accelerate the discovery of antibiotics. Moreover, actinobacteria living in the QTP holds significant potential for the exploration of new antibiotics and deserve in-depth research.

## 2. Materials and methods

### 2.1. Sample collection

**Figure 2:**
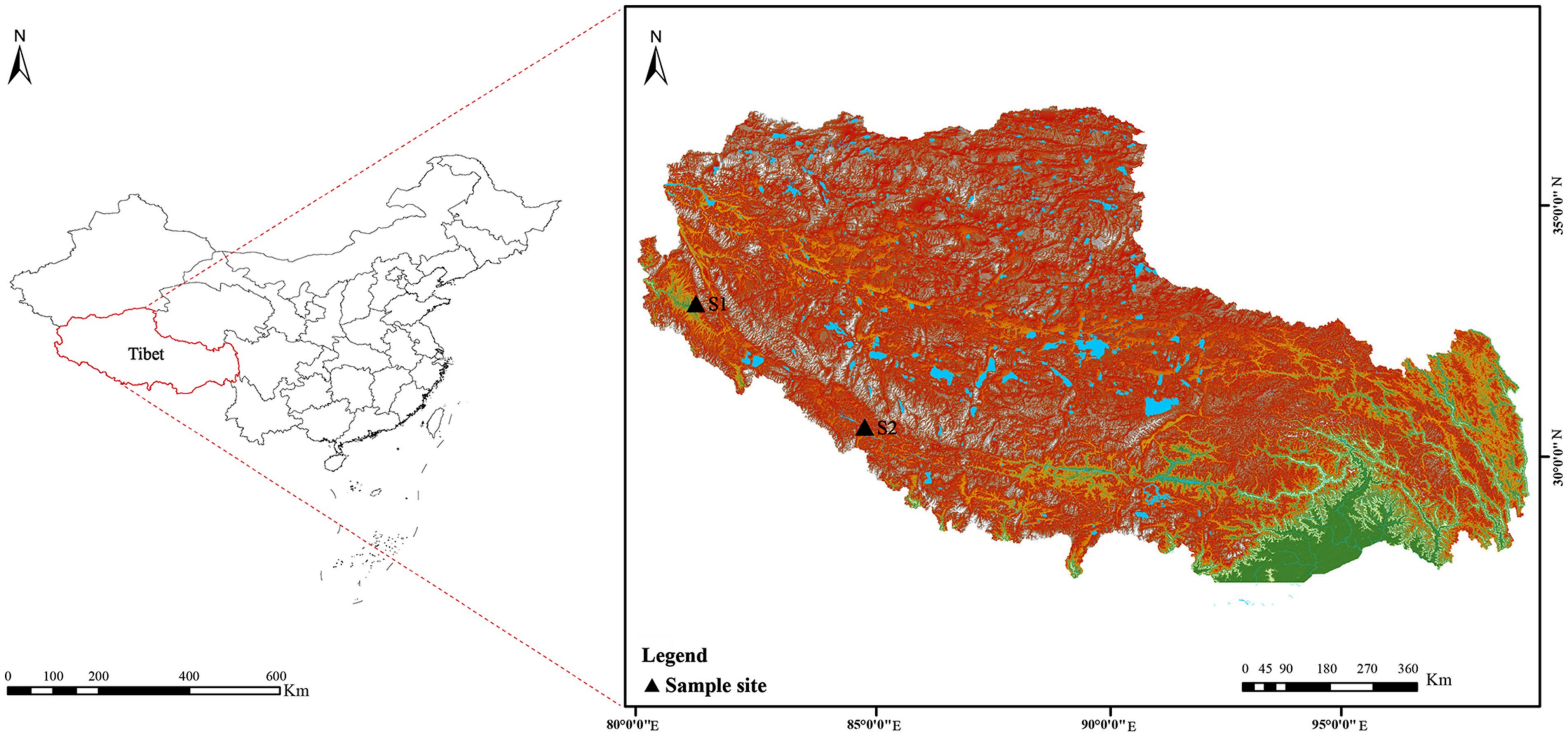
Geographic distribution of the sampling sites (S1–S2) on the Western Qinghai-Tibet Plateau, Tibet Autonomous Region, China.

Soil samples (S1 and S2) were collected from Tibet in July 2020. The details of the sample collection location were as follows (Figure 2): S1 was collected from the Zada County (latitude 31°34′21″ N, longitude 79°53′56″ E, elevation 4,450 m); S2 was collected from the Zhongba County (latitude 29°43′18″ N, longitude 84°2′30″ E, elevation 4,567 m). Samples, collected at a 5–15 cm depth, were packed into 50 ml sterile Falcon tubes and kept at 4°C until further analysis. Prior to isolation, two soil samples were air-dried at room temperature for 8 h in a laminar flow cabinet.

### 2.2. Actinobacteria isolation and preservation

Actinobacterial strains were isolated using 10 different isolation media (Supplementary Table S1). To minimize the contamination of Gram-negative bacteria and fungi, nalidixic acid (20 μg/ml), cycloheximide (50 μg/ml), and potassium dichromate (50 μg/ml) were added to each medium. Soil suspension of two samples from 10^−2^ to 10^−4^ was dispersed onto selective media by the standard serial dilution plating technique as described by Li F. et al. (2019). After incubation at 28°C for 2–4 weeks, colonies with visibly diverse morphologies were picked up, streaked, and sub-cultured to ISP 2 media to achieve pure colonies (Liu et al., 2021). The pure cultures were maintained on ISP 2 agar slants at 4°C and 20% (v/v) glycerol suspensions at −80°C.

### 2.3. Taxonomic identification and phylogenetic analysis of isolates

**Figure 3:**
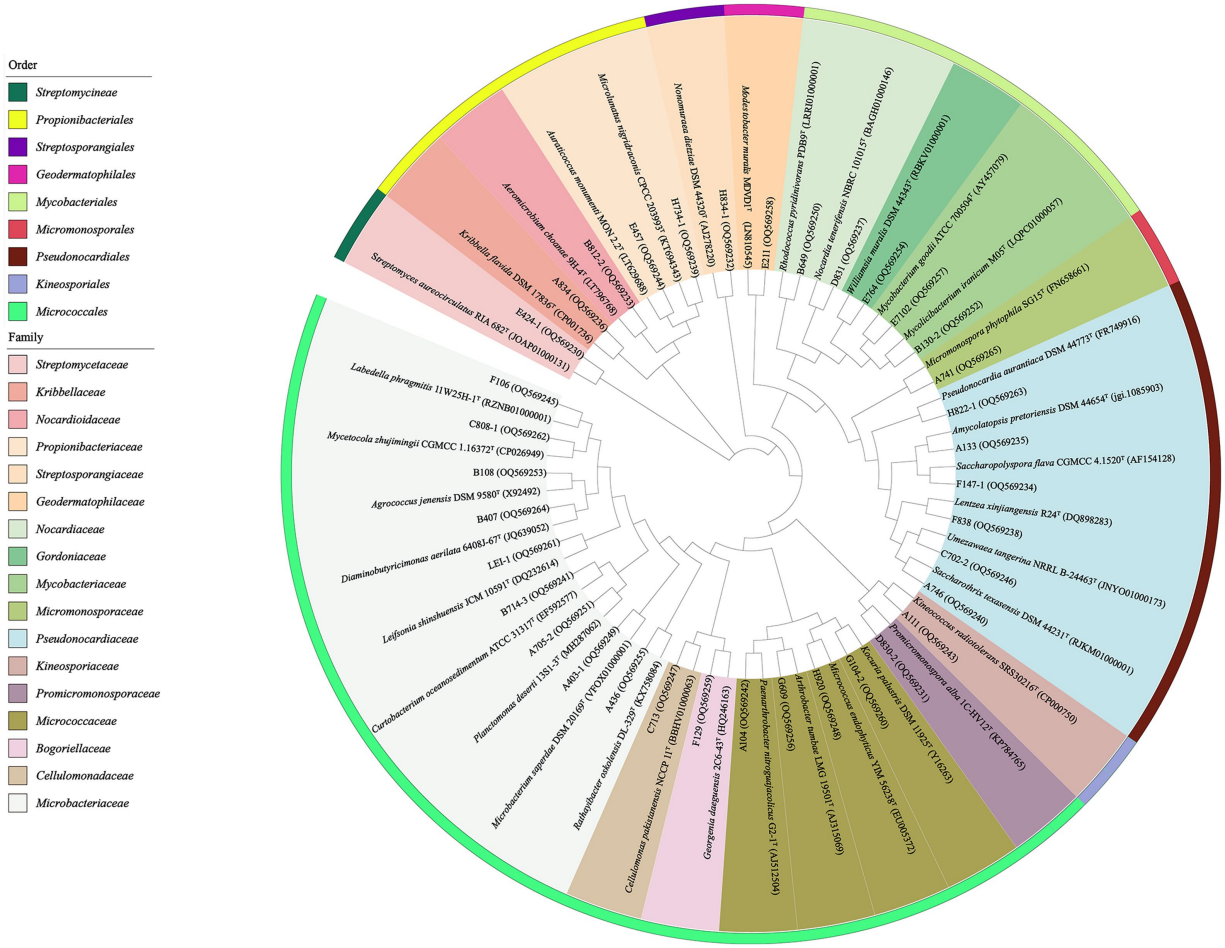
Phylogenetic tree based on the 16S rRNA gene sequences using neighbor-joining method with 1,000 bootstrap replications for 36 representative actinobacterial strains isolated and their nearest neighbors in EZBiocloud.

The primary taxonomical identification of pure isolates was accomplished by analysis of 16S ribosomal RNA (16S rRNA) gene sequencing, as described by Li et al. (2007). The 16S rRNA genes sequences were amplified by PCR using two universal primers, 27F (5′-AGAGTTTGATCMTGGCTCAG-3′) and 1492R (5′-GGTTA CCTTGTTACGACTT-3′; Liu et al., 2014). The amplified products were sequenced using the ABI PRISMTM 3730XL DNA Analyzer (ABI, USA). The preliminary taxonomy of strains was determined by comparing their 16S rRNA gene sequences with data from EzBioCloud’s Identify service^1^ and the BLAST tool in the GenBank NCBI database (Yoon et al., 2017). The corresponding sequences of the closest type strains for phylogenetic tree construction were retrieved from the EZBiocloud. Subsequently, using the MEGA (molecular evolutionary genetics analysis) program, version 7.0, the phylogenetic tree was constructed based on the neighbor-joining algorithm (Saitou and Nei, 1987). Multiple alignments were completed through the Clustal_X tool in MEGA version 7.0 (Kumar et al., 2016). Bootstrap replications of 1,000 were assessed for the evolutionary distances (Kimura, 1980). The 16S rRNA gene sequences of antibacterial strains and representative strains for the phylogenetic tree obtained in this study were deposited in the GenBank under accession numbers in the range of OQ569230-OQ569340 (Figure 3; Supplementary Table S4). The 16S rRNA gene sequences of potential new species were deposited in the GenBank under accession numbers of OR470596-OR470601 (Supplementary Table S3).

### 2.4. Antimicrobial activity assay

Through dereplication of strain morphology and the 16S rRNA gene sequences, 152 out of 406 strains were selected as representative strains for antimicrobial testing. Each strain was inoculated into the sterilized 100 ml cottonseed powder broth (containing, per 1 L distilled water: 5.0 g yeast extract, 5.0 g glucose, 10.0 g maltose, 10.0 g cottonseed powder, 20.0 g soluble starch, pH 7.2) in a 500 ml Erlenmeyer flask, then incubated at 28°C in a rotary shaker with 180 rpm for 5–10 days. The harvested broth was centrifuged at 4,500 rpm and extracted three times in parallel with equal volumes of ethyl acetate. Afterward, the crude extract of the organic solvent phase (ethyl acetate extract) was obtained through concentration process under vacuum. Based on the paper-disk diffusion method, the ethyl acetate extract was dissolved in 1 ml of methanol for antimicrobial evaluation. Briefly, 60 μl of sample was dropped on a sterile paper disc with a diameter of 5 mm, dried in a biosafety hood, and then transferred onto a Mueller-Hinton (MH) agar plate with an indicator strain, the transparent zone in a plate was observed by incubation at 37°C for 24 h. Meanwhile, the 60 μl of pure methanol and the 10 μl of levofloxacin (0.1 mg/ml) were used as a negative and a positive control, respectively. The antimicrobial activity was evaluated by measuring the diameter of the inhibition zone on the agar plates after incubating 24 h. Twelve “ESKAPE” strains were chosen for antibacterial activities evaluation, including *Enterococcus faecalis* (ATCC 33186 and 310682), *Staphylococcus aureus* (ATCC 29213 and ATCC 33591), *Klebsiella pneumoniae* (ATCC 10031 and ATCC 700603), *Acinetobacter baumannii* (2799 and ATCC 19606), *Pseudomonas aeruginosa* (ATCC 27853 and 2774), and *Escherichia coli* (ATCC 25922 and ATCC 35218). Each of the six groups of pathogenic bacteria mentioned above was composed of one drug-sensitive strain and one drug-resistant strain, as reported by Liu et al. (2021). All the indicator strains were stored in the Institute of Medicinal Biotechnology, Chinese Academy of Medical Sciences.

### 2.5. Antimicrobial mechanism assay

The “pDualrep2” system was used to evaluate the mechanism of antimicrobial action of the ethyl acetate extract of strain with antimicrobial activity. This system is based on hypersensitive strain *E. coli* JW5503 (*Δ*tolC) transformed with “pDualrep2” plasmid (Baba et al., 2006), which allowed to sort out suppressors of protein synthesis or SOS-response induces (Osterman et al., 2016). In short, 100 μl of DMSO was added into each of the dried ethyl acetate extract as a sample for the antimicrobial mechanism detection. About 2 μl of the sample solution was dropped on an agar plate containing the reporter strain *E. coli* JW5503 and incubated overnight at 37°C. The agar plate was scanned using the ChemiDoc Imaging System (Bio-Rad Laboratory, USA). This system consisted of two channels, “Cy3-blot” (553/574 nm, green pseudocolor) fluorescent for Turbo red fluorescent protein (TurboRFP) and “Cy5-blot” (588/633 nm, red pseudocolor) for Katushka2S fluorescence. Translation inhibitors triggered the induction of Katushka2S expression, while TurboRFP was upregulated by SOS response. Levofloxacin (Lev, 50 μg/ml, 1 μl) and erythromycin (Ery, 5 mg/ml, 1 μl) were utilized as positive controls for DNA and protein translation inhibitors, separately.

### 2.6. UPLC-QTOF-MS/MS analysis

The ethyl acetate extract from the strain A133 was analyzed through UPLC-HRESI-MS/MS (Waters Xevo G2-XS QTOF, Waters, United States) fitted with the ACQUITY UPLC BEH C18 column (2.1 × 100 mm, 1.7 μm). The sample was dissolved in methanol and filtered, then subjected to gradient elution in an acetonitrile-water solution system at a flow rate of 0.3 ml/min. For chromatographic separation, the gradient elution method was 10% acetonitrile for 1 min, then 10 to 100% in a linear gradient over 17 min, and finally eluted under 100% acetonitrile for 2 min. The PDA detector for monitoring UV absorption was set from 190 to 600 nm. MS spectra were acquired through both the data-independent acquisition mode (MSE) and the data-dependent acquisition mode (DDA; Liu et al., 2021). The following acquisition settings were used for mass data capture in the positive mode: the source temperature and desolvation temperature were set to 100°C and 250°C, respectively; the sampling cone voltage at 40 eV; the capillary voltage at 2 kV; the source offset voltage at 80 eV; the high-purity nitrogen was used as the nebulizer and auxiliary gas; the cone gas was set to 30 L/h; meanwhile, the flow rate of desolvation gas was 600 L/h. Mass accuracy was maintained by using a lock spray with leucine-enkephalin (positive ion mode [M + H]^+^ = 556.2771 Da) as reference with a concentration of 200 pg/ml and a flow rate of 10 μl/min. MSE provided well-informative mass spectrometry data acquisition in positive ion mode. The mass-to-charge ratio (m/z) in the acquisition ranged from 100 to 1,500 Da, and the collision energy ranged from low (4 V) to high (20–40 V). DDA acquisition was conducted in a positive ion mode with a complete MS survey scan at a scanning time of 0.2 s in the range of 100–1,600 Da, while MS/MS was carried out in the mass range of 50–1,600 Da with the same scanning time. The five highest-intensity ions were further selected for MS/MS fragmentation spectra. Data were collected and analyzed using MassLynx V4.1 software (Waters, United States). Methanol and the extracts from the uninoculated medium were also examined as the control.

### 2.7. Molecular networking and dereplication analysis of microbial secondary metabolites

The raw DDA data was converted to 32-bit mzML format with MSConvert and uploaded to the online workflow on the GNPS website to construct a classical molecular network (Chambers et al., 2012; Wang et al., 2016). The molecular network obtained on basis of the following parameters: set the precursor mass tolerance to 0.1 Da, and set the MS/MS fragment ion tolerance to 0.1 Da; the network was then established based on edges filtered with a minimum cosine score of 0.6 and more than 4 matched fragment ions; in addition, an edge between 2 nodes remained in the network if and only if each node appears in the top 10 most similar nodes of each other; the size of a molecular family was set to no more than 100, and removed inappropriate scoring edges and until the size of the molecular family did not exceed this threshold; the spectra were searched against GNPS’ spectral libraries with the same method as the input data. The data clusters obtained were analyzed and dereplicated in advanced analysis tools including Network Annotation Propagation (NAP) and Dereplicator + (Wang et al., 2016; Kang et al., 2018; Mohimani et al., 2018; da Silva et al., 2022). The resulting molecular network data was visualized via Cytoscape 3.9.1 (Shannon et al., 2003). Manual analysis was performed in Natural Product Atlas (van Santen et al., 2019), Streptome DB database (Moumbock et al., 2021), and related literatures to improve the dereplication.

### 2.8. Identification of genome sequencing and biosynthesis gene clusters

The genomic DNA extraction of the strain A133 was performed using the method previously described by Li F. N. et al. (2019). The draft genome sequencing and analysis were conducted by OE Biotech Co., Ltd. (Shanghai, China). The bioinformatics tool antiSMASH 7.0.0 (Blin et al., 2023) was used to examine the biosynthetic gene clusters and assess the potential of strain A133 for bioactive secondary metabolites biosynthesis. The whole-genome sequence of the strain A133 was deposited in the NCBI GenBank under accession number JAVHJU000000000.

### 2.9. Isolation and identification of secondary metabolites from the strain A133

Based on its antibacterial activities and the underlying mechanism, the strain A133 was prioritized for further chemical analysis. Strain A133 was inoculated into a 100 ml of ISP2 broth medium in a 500 ml Erlenmeyer flask, and cultured at 28°C in an orbital shaking incubator at 180 rpm for 3 days to obtain seed liquid. The seed liquid was inoculated into a 1 L cottonseed powder broth medium for scaled-up fermentation under the same cultural conditions for 7 days. Totally, 150 L fermentation broth was centrifuged at 4,100 rpm for 25 min at 20°C. Three parallel extractions of the supernatant were performed using equivalent volumes ethyl acetate. Under the vacuum state, the organic phase was evaporated to obtain a crude extract (1.3 g). The ethyl acetate extract was fractionated using the reversed-phase flash chromatography (Biotage, Sweden) equipped with RP-C18 column (ODS-A, YMC, 100 g) and eluted with a linear gradient solvent system of methanol–water (methanol: 20–100%) at a flow rate of 10 ml/min to yield five fractions (Fr.1 to Fr.5). With the assistance of LC–MS analysis, Fr.3 was separated by methanol on Sephadex LH-20 column chromatography, and the target fractions were further subjected to normal-phase chromatography using a silica column (20 g) with a linear gradient system of 50:1 to 1:1 of chloroform-methanol to yield to three subfractions (F_C-1_ to F_C-3_); F_C-1_ was firstly subjected to Sephadex LH-20 column chromatography and eluted with methanol, then purified through the semi-preparative high-performance liquid chromatography (HPLC, Agilent 1,200, Agilent Technologies Inc., United States) over a C18 column (Agilent ZORBAX SB-C18, 9.4 × 250 mm, 5 μm) using the isocratic 32% acetonitrile-water solution at a flow rate of 2.0 ml/min to yield target compounds **1** (6.7 mg, t_R_ = 61 min) and **2** (2.3 mg, t_R_ = 52 min); F_C-2_ was subjected to flash chromatography using RP-C18 column (20 g) and a gradient of methanol–water (methanol: 60–75%) at a flow rate of 5 ml/min to generate a mixture with compounds **3** and **4**, the mixture was subsequently purified by HPLC equipped with a C18 column using the isocratic 40% acetonitrile-water solution at a flow rate of 2.0 ml/min to get target compound **3** (7.5 mg, t_R_ = 57 min) and a semi-purified compound **4**, compound **4** (3.3 mg, t_R_ = 81 min) was obtained through the HPLC over the C18 column using the isocratic 37% acetonitrile-water solution at a flow rate of 2.0 ml/min; F_C-3_ was firstly subjected to flash chromatography over the RP-C18 column (20 g) using a linear gradient solvent system of methanol–water (methanol: 50–65%) as the mobile phase, and then further purified by HPLC equipped with C18 column. After eluted with isocratic 45% acetonitrile-water solution at a flow rate of 2.0 ml/min, compound **5** (5.4 mg, t_R_ = 79 min) was obtained. The chemical structures of the 5 compounds obtained were elucidated based on data collected from NMR spectroscopy (Brucker Avance III 600 spectrometer, Germany) and HRESI-MS/MS mass spectral data (Xevo G2-XS QTOF, Waters, United States).

## 3. Results

### 3.1. Biodiversity and novelty of cultivable actinobacteria from soil on the Western Qinghai-Tibet Plateau

**Figure 4:**
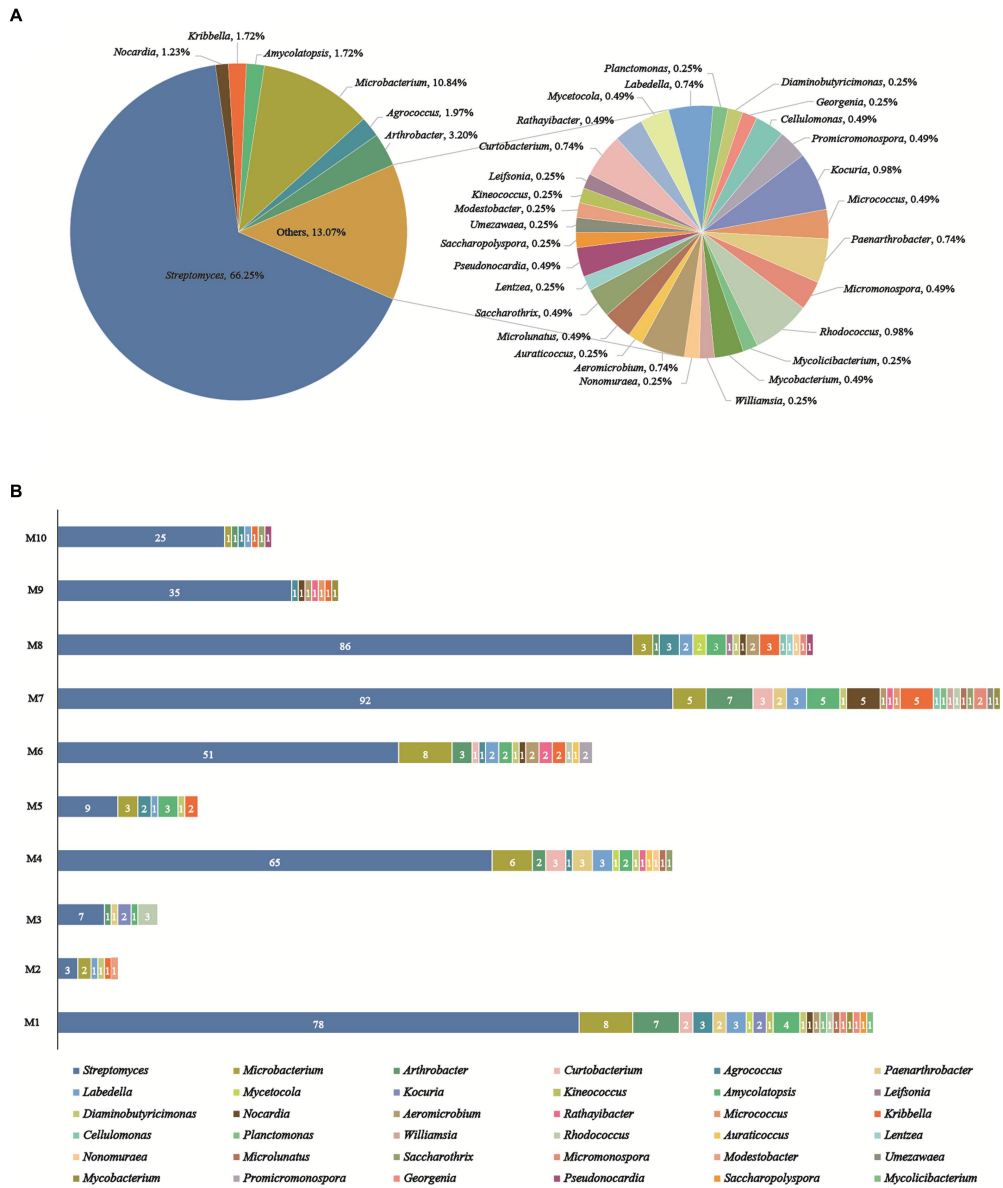
Diversity of cultivable actinobacteria recovered from the soils on the Western Tibetan Plateau. **(A)** Percentage of recovered isolates belonging to different genera. **(B)** Actinobacterial genera distribution in 10 selective culture media.

A total of 406 actinobacteria were recovered from two soil samples collected from the Western Qinghai-Tibet Plateau, and identified through analysis of the partial 16S rRNA gene sequencing (> 750 bp). The 406 actinobacteria were categorized into 36 genera in 17 families of 9 orders. To construct and analyze the phylogenetic tree (Figure 3), representative 16S rRNA gene sequences from different genera were chosen. Notably, analysis of the 16SrRNA gene similarity indicated that these actinobacteria were distributed in 36 genera (Figure 4A): *Streptomyces* (66.3%, 269 strains) was the most dominant genus comprising 66.3% of the total isolates; followed by *Microbacterium* (10.8%, 44 strains), *Arthrobacter* (3.2%, 13 strains), *Agrococcus* (2.0%, 8 strains), both *Amycolatopsis* and *Kribbella* (1.7%, 7 strains), *Nocardia* (1.2%, 5 strains), both *Rhodococcus* and *Kocuria* (1.0%, 4 strains); the remaining 27 genera had less than 1% abundance. Moreover, their relative abundance at the order level was illustrated in Supplementary Table S2: *Streptomycineae* or *Kitasatosporales* (Göker et al.,) was the most abundant order comprising 66.3% (269 strains) of the total number of actinobacteria, followed by *Micrococcales* (22.7%, 92 strains), *Pseudonocardiales* (3.5%, 14 strains), *Mycobacteriales* and *Propionibacteriales* (both 3.2%, 13 strains each); *Micromonosporales* (0.5%, 2 strains), as well as *Streptosporangiales*, *Geodermatophilales*, and *Kineosporiales* (0.2%, 1 strain each).

As illustrated in Figure 4B, the utilization of 10 distinctive isolation media considerably impacted the abundance and diversity of cultivable strains. R2A (M1) and Glycerol-Casein (M7) media yielded the highest diversity of actinobacteria (22 genera). The Glycerol-Casein (M7) medium yielded the greatest number of rare actinobacteria (49 strains, 34.8%), while 21 rare genera were identified in the M1 (44 strains, 36.1%). Modified HV (M8) medium produced 113 strains in 17 genera, including 16 rare genera (27 strains, 23.9%). Two different isolation media, including Modified ISP 2 (M4) and CMKA (M6) media, both yielded 15 genera, while the number of rare actinobacteria isolates obtained from M6 (29 strains, 36.3%) was more than M4 (27 strains, 29.3%). Among the other media used, Modified Proline (M2), Raffinose-Histidine (M5), Glyceryl-Arginine (M3), Propionate-Casein (M9), and Humic Acid (M10) media all yielded actinobacteria with no more than 9 genera in diversity, meanwhile, 5, 6, 5, 7, and 7 rare genera were obtained from M2 (6 strains, 66.7%), M5 (12 strains, 57.1%), M3 (8 strains, 53.3%), M9 (7 strains, 16.7%), and M10 (7 strains, 21.9%), respectively. Among all media, modified Proline (M2) medium had the lowest diversity and abundance, including 9 strains in 6 genera. The genus of *Streptomyces* was dominant and recovered from 10 culture media. Besides, rare actinobacteria appeared in all isolation media, especially in M1, M4, M6, M7, and M8.

Based on comparative analysis using the EzBiocloud database, 6 strains displayed less than 98.65% similarities in their16S rRNA gene sequences compared to validly described species (Kim et al., 2014; Supplementary Table S3). These results suggested that these isolates could be regarded as primary candidates for novel taxon. These 6 putative novel isolates were initially affiliated into 3 families, including *Microbacteriaceae* (2 strains), *Streptomycetaceae* (3 strains), and *Streptosporangiaceae* (1 strain).

### 3.2. Antimicrobial activity

**Figure 5:**
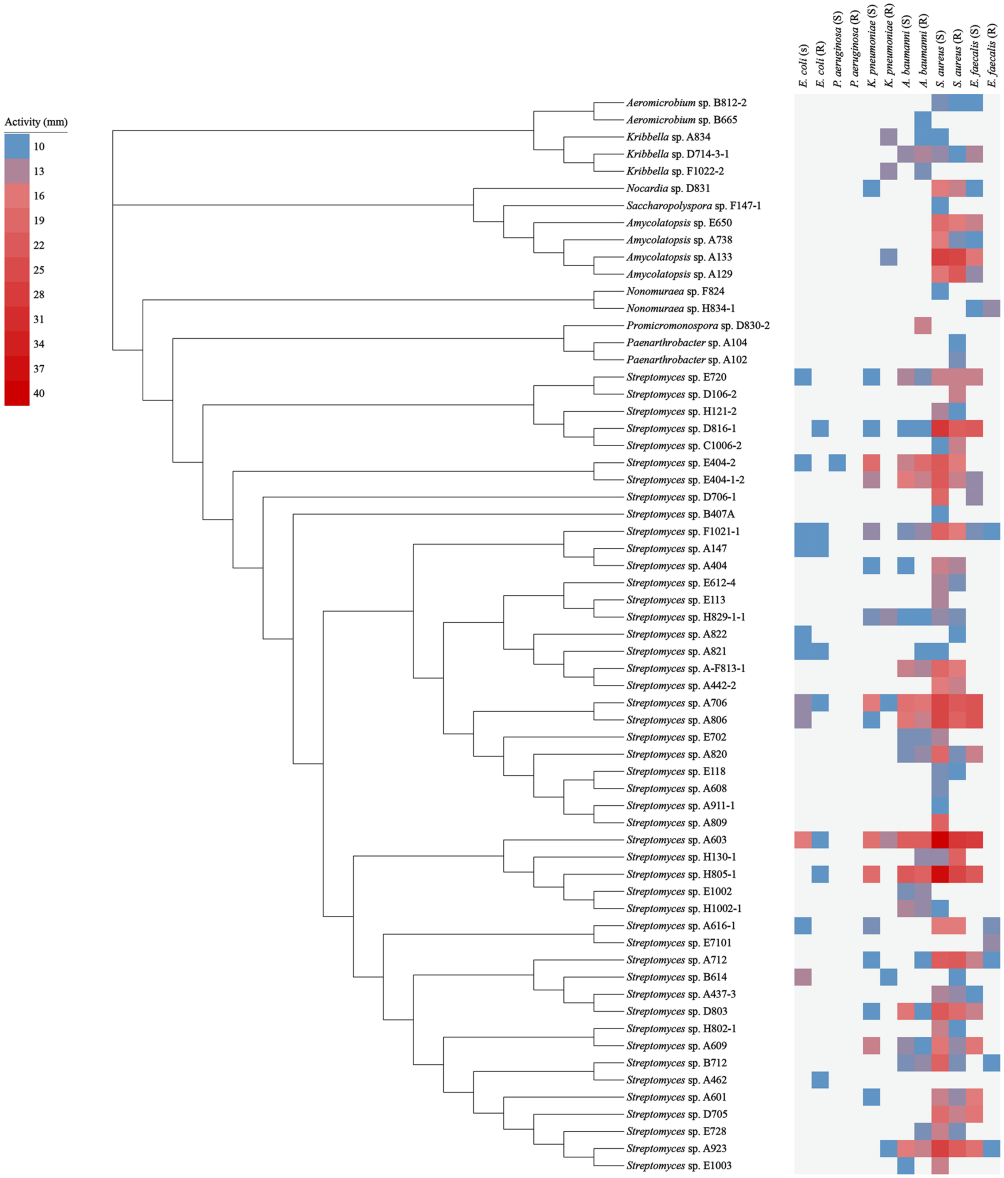
Antimicrobial activity of 63 representative strains. The blue squares represent the smallest inhibition circle of 10 mm; the light to dark red squares represent increasing inhibition activity with a maximum inhibition circle of 40 mm.

In the antibacterial assay against “ESKAPE” pathogenic bacteria, 63 out of 152 tested strains exhibited antibacterial activity against at least one of the pathogens, as shown in Figure 5 and Supplementary Table S4. These bioactive strains were belonged to 9 genera, including *Streptomyces* (47 strains), *Nonomuraea* (2 strains), *Amycolatopsis* (4 strains), *Kribbella* (3 strains), *Aeromicrobium* (2 strains), *Nocardia* (1 strain), *Promicromonospora* (1 strain), *Paenarthrobacter* (2 strain), and *Saccharopolyspora* (1 strain). Specifically, 32 strains demonstrated antimicrobial activity against both Gram-positive and Gram-negative bacteria; while, 25 and 6 strains displayed antimicrobial activities specifically against Gram-positive and Gram-negative bacteria, respectively.

For the isolates against Gram-positive pathogens, the amounts of bioactive isolates against *S. aureus* (55 isolates) were evidently more than those against *E. faecalis* (28 isolates). While for the isolates against Gram-negative pathogens, there were more antibacterial isolates against *A. baumannii* (30 isolates) compared to those against *K. pneumonia* (22 isolates) and *E. coli* (14 isolates). Only 1 isolate was found to show antibacterial activity against *P. aeruginosa*.

Besides, in terms of inhibitory activity against drug-sensitive pathogens: the number of isolates against drug-sensitive *S. aureus*, *E. faecalis*, *A. baumannii*, *K. pneumonia*, *E. coli*, and *P. aeruginosa* was 50, 25, 22, 17, 11 and 1, respectively, while the corresponding number against drug-resistant pathogens above mentioned was 42, 7, 28, 8, 8 and 0, respectively.

It was worth highlighting that, amongst all of the bioactive non-*Streptomyces* strains, the strain A133 demonstrated the conspicuously antimicrobial activity, including the most potent antagonistic activity against *S. aureus* (S, 27 mm), followed by *S. aureus* (R, 26 mm), *E. faecalis* (S, 16 mm) and *K. pneumoniae* (R, 11 mm).

### 3.3. Determination of antibacterial mechanism

**Figure 6:**
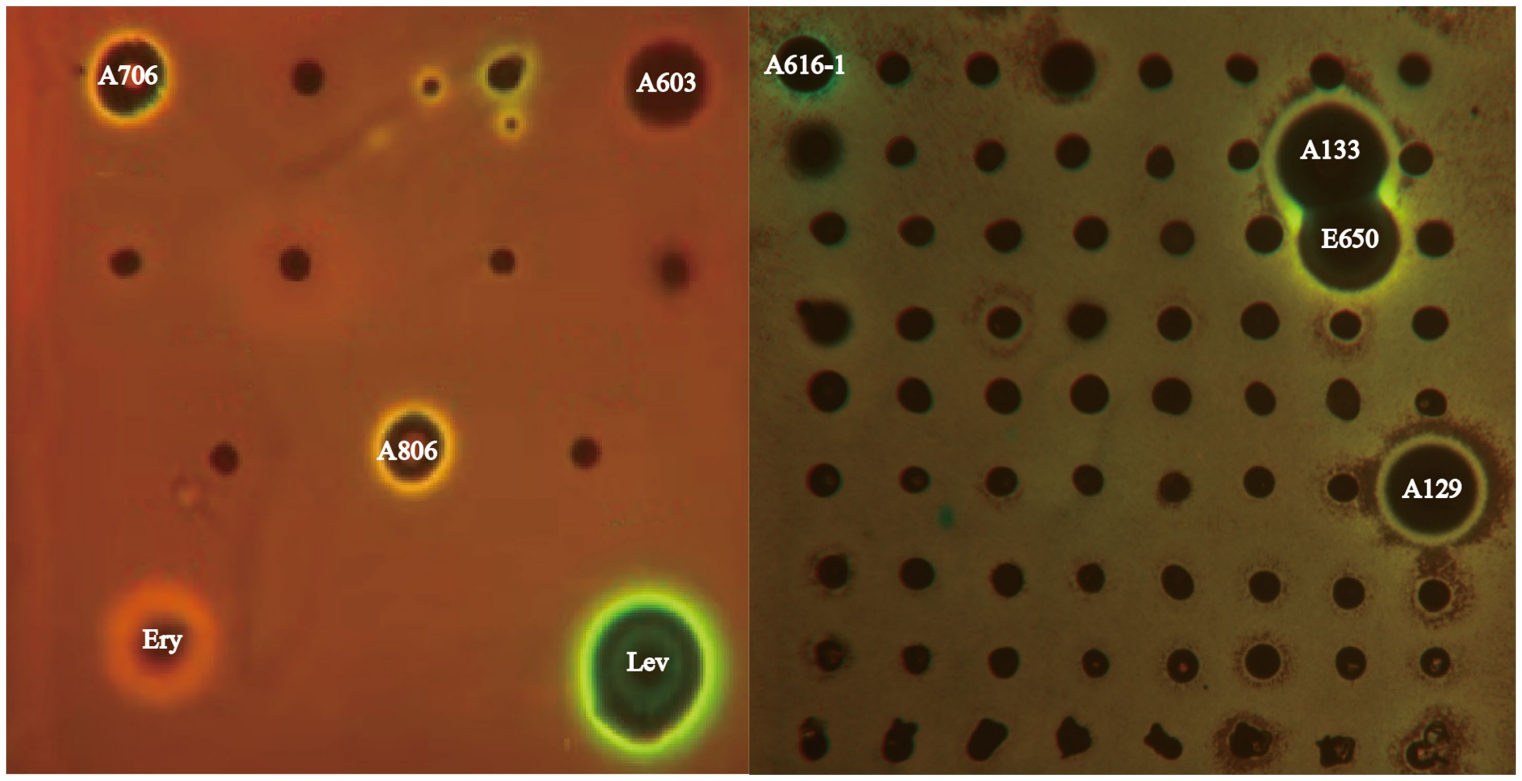
The dual-fluorescent reporter system “pDualrep 2.” Spots of erythromycin (E), levofloxacin (L), and tested samples were placed on the surface of an agar plate coated with *E. coli* JW5503 reporter transformed with the pDualrep2 reporter plasmid. The plate was scanned under the at 553/574 nm and 588/633 nm channels to detect red fluorescent protein (green pseudocolor) and Katushka2S (red pseudocolor) fluorescence, respectively.

Sixty-three bioactive strains are potential candidates to produce antibiotics with various mechanisms of action. To distinguish strains with different antibacterial mechanisms, extracts from culture broth of 63 bioactive strains were screened by the double fluorescent protein reporter “pDualrep2” system, which is a highly sensitive screening model for probe of compounds that inhibit protein translation or DNA biosynthesis. As illustrated in Figure 6, extracts of three *Streptomyces* strains (A603, A706 and A806) induced expression of reporter fluorescent protein Katushka2S, acting as typical inhibitors of protein translation as the erythromycin did. Meanwhile, three extracts of *Amycolatopsis* (A133, E650 and A129) strains and one extract of *Streptomyces* (A616-1) strain induced expression of RFP reporter, triggering SOS response as the levofloxacin did. Based on the activity reported, strain A133 not only showed a remarkable antibacterial activity, but also induced a SOS response mechanism similar to levofloxacin. Thus, to uncover the bioactive secondary metabolites from the extract of the strain A133, a series of omics studies were carried out.

### 3.4. Identification of biosynthetic gene clusters for the strain A133

To predict the potential secondary metabolites with antibacterial activities produced by the strain A133, the draft genome of strain A133 was sequenced, which was featuring a single 10,054,484 bp chromosome with 71.96% GC content. After analysed by antiSMASH, 34 gene clusters were found to be associated with secondary metabolites (Supplementary Table S5), and three of them belonged to the known clusters for ectoine, geosmin, and detoxins with 100% similarity, one resembled the cluster of scabichelin with 80% similarity, as well as the cluster 23 showed 73% similarity to cluster of rifamorpholines, a group of rifamycins. The other clusters exhibited similarity less than 60% with the known clusters, including arixanthomycins, amycolamycins, isorenieratene, etc.

### 3.5. Metabolites identification of *Amycolatopsis* sp. A133 using MS/MS-based molecular networking

**Figure 7:**
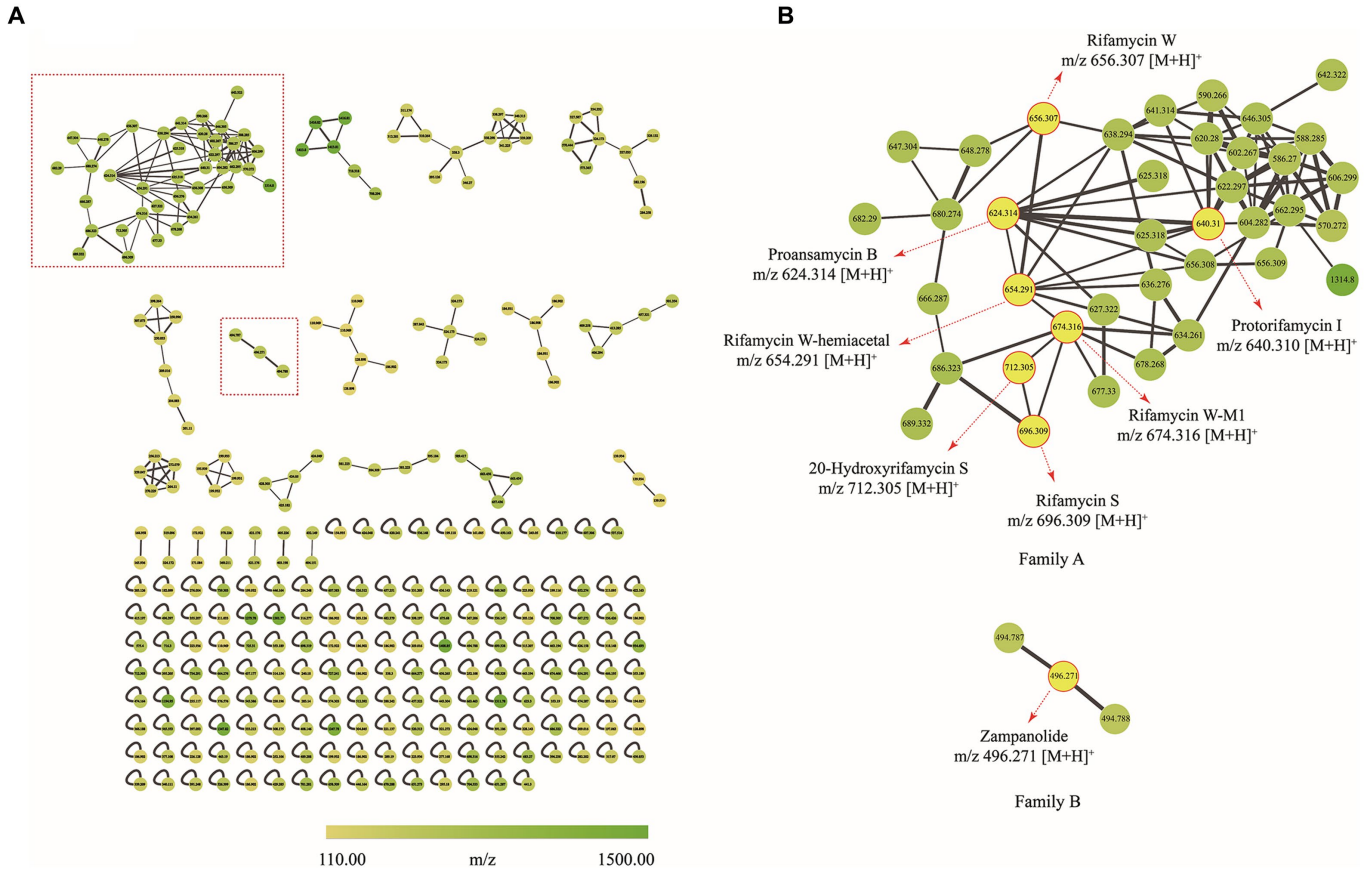
Molecular network of secondary metabolites from the strain A133. **(A)** Whole molecular network realized using MS/MS data from of strain A133. **(B)** Two identified families (family A and family B) in molecular network. Each node represents m/z value of the parent ion and the edge thickness indicates the cosine score similarity. Cluster of rifamycins was assigned as family A; Cluster of zampanolides was assigned as family B.

To decipher the bioactive secondary metabolites from the strain *Amycolatopsis* sp. A133, the ethyl acetate extract was subjected to UPLC-QTOF MS/MS-based molecular networking analysis, the acquired fragmented data was grouped into clusters and then compared with the existing data in the GNPS database to achieve dereplication. Thus, a molecular network consisting of 292 nodes that covered a broad range of parent ions from m/z 110 to 1,500, was generated (Figure 7A). Among them, 133 nodes were clustered into 23 clusters (nodes ≥2) with 192 edges, leaving out the solvent blank and growth medium control. It was inevitable to find multiple nodes corresponding to the same compound, given that nodes reflecting the same chemical entity in a molecular network may represent its molecular mass in different mono- or multi-charged adducts. After a thorough and rigorous comparison and matching process in MS/MS spectral database and advanced analysis tools, several nodes were identified to be compounds from two families (family A and family B; Figure 7B).

Family A was annotated as rifamycins, which was in line with the biosynthetic gene clusters analysis result. The family A was comprised of 38 spectral nodes. The precursor masses within this family ranged from m/z 570 to 1,314 Da. Apart from rifamycin W (**1**, C_35_H_46_NO_11_, m/z [M + H]^+^ 656.3074) annotated directly by GNPS analysis, the other six rifamycin derivatives were preliminarily identified after series processing, including comparing their precursor ion m/z values, fragmentation data, and UV data with several database and related literatures. Specifically, such derivatives included protorifamycin I (**2**, C_35_H_46_NO_10_, m/z [M + H]^+^ 640.3115), rifamycin W-M1 (**3**, C_35_H_48_NO_12_, m/z [M + H]^+^ 674.3159), proansamycin B (**4**, C_35_H_45_NO_9_, m/z [M + H]^+^ 624.3193), rifamycin S (**5**, C_37_H_46_NO_12_, m/z [M + H]^+^ 696.3023), 20-hydroxyrifamycin S (m/z [M + H]^+^ 712.2969; Shi et al., 2019), and rifamycin W-hemiacetal (m/z [M + H]^+^ 654.2915; Ansgar et al., 2002; Supplementary Table S6).

Family B contained 3 nodes (m/z 494 to 496 Da), and the node (m/z [M + H]^+^ 496.271) was annotated as zampanolide (C_29_H_37_NO_6_) as shown in Supplementary Table S6. Zampanolide, a promising microtubule-stabilizing agent, was first isolated from marine sponge *Fasciospongia rimasa* in 1996 (Tanaka and Higa, 1996; Field et al., 2012; Chen and Kingston, 2014). In 2018, four members of zampanolides, named zampanolides B–E, were also isolated from the marine sponge *Cacospongia mycofijiensis* (Taufa et al., 2018). The molecular formulas of zampanolides B–D were identical to that of zampanolide, whereas the molecular formula of zampanolide E was C_29_H_39_NO_6_ ([M + Na]^+^ 520.2674; Taufa et al., 2018).

Based on omics analysis utilizing molecular networking and biosynthetic gene clusters, it was crucial to isolate and precisely identify the annotated secondary metabolites.

### 3.6. Secondary metabolites isolation and identification

With the assistance of LC–MS analysis, five rifamycin analogues were isolated and purified as target compounds from the ethyl acetate extract. Their structural elucidation was accomplished on the basis of NMR and HR-ESI-MS data (Supplementary Figures S1–S10; Supplementary Table S7), along with a comparison with the published literatures. In line with the results of the molecular networking analysis, five target compounds (Figure 1) were indeed rifamycin derivatives, including rifamycin W (**1**; Ghisalba et al., 1978), protorifamycin I (**2**; Ghisalba et al., 1978), rifamycin W-M1 (**3**; Shi et al., 2021), proansamycin B (**4**; Ghisalba et al., 1980), and rifamycin S (**5**; Ghisalba et al., 1980). As reported, rifamycin W-M1 was first isolated from an *Amycolatopsis* mutant strain, the rif-orf5 gene knock-out mutant (Shi et al., 2021). In this study, rifamycin W-M1 was first isolated from an ungenetically manipulated *Amycolatopsis* strain. In addition, the molecular networking of family A revealed some nodes with different parent ions have not been found in the published literatures and GNPS database, which could be concluded that some new members of rifamycins were produced by the strain A133.

## 4. Discussion

The Western Qinghai-Tibet Plateau, particularly the Ali region, is a hard-to-reach area with multiply extreme environmental factors. To our knowledge, studies on culturable actinobacteria and their bioactive secondary metabolites in this region are relatively less. Ma et al. (2020) isolated 50 *Streptomyces* strains from near-root soils in the Gaize County, of which 13 strains showed inhibitory effects against human pathogens including *E. coli*, *S. aureus*, *C. albicans*, and *P. aeruginosa*. Huang et al. (2017) analysed culturable actinobacteria retrieved from 14 soil samples in the Ali, Naqu, and Haixi region. Their findings revealed the presence of 255 isolates distributed in 23 genera, including *Actinomadura*, *Actinomycetospora*, *Agromyces*, *Arthrobacter*, *Brevibacterium*, *Citricoccus*, *Curtobacterium*, *Georgenia*, *Jiangella*, *Kribbella*, *Kocuria*, *Lentzea*, *Microbacterium*, *Micromonospora*, *Mycobacterium*, *Nocardia*, *Nonomuraea*, *Pseudonocardia*, *Promicromonospora*, *Rhodococcus*, *Saccharothrix*, *Streptomyces*, and *Streptosporangium*. Li et al. (2018) also investigated the actinobacterial communities from soils in the color desert of Dengpa. They identified 231 actinobacteria, which included *Streptomyces*, *Nocardioides*, *Lentzea*, *Kocuria*, *Nocardia*, *Saccharothrix*, and *Kineococcus*. Several of these strains demonstrated the ability to inhibit certain pathogens and had antioxidant capacity towards hydroxyl and oxygen-free radical scavenging. Moreover, 9 cytotoxic secondary metabolites were isolated from *Streptomyces dengpaensis* XZHG99T, 3 of which were new angucyclines (Bao et al., 2018).

In this study, we investigated the diversity of actinobacteria in two soil samples, ultimately identifying 406 isolates affiliated with 36 different genera. The results revealed that some genera, including *Streptomyces*, *Micromonospora*, *Rhodococcus*, *Nocardia*, *Mycobacterium*, *Nonomuraea*, *Kribbella*, *Saccharothrix*, *Lentzea*, *Microbacterium*, *Curtobacterium*, *Georgenia*, *Promicromonospora*, *Kocuria*, *Kineococcus*, *Pseudonocardia*, and *Arthrobacter*, were detected in both our study and earlier research mentioned above. Additionally, other 19 rare genera, including *Mycolicibacterium*, *Williamsia*, *Aeromicrobium*, *Auraticoccus*, *Microlunatus*, *Amycolatopsis*, *Saccharopolyspora*, *Umezawaea*, *Modestobacter*, *Agrococcus*, *Leifsonia*, *Rathayibacter*, *Labedella*, *Planctomonas*, *Diaminobutyricimonas*, *Cellulomonas*, *Micrococcus*, *Mycetocola*, and *Paenarthrobacter*, were isolated in our study.

In our research, over half isolates (269 out of 406) were identified as *Streptomyces*, which is consistent with previous publications (Huang et al., 2017; Li et al., 2018; Ma et al., 2020). This phenomenon is well-documented by the high adaptability of *Streptomyces* to various harsh environmental conditions (Ma et al., 2020). To withstand extreme conditions, *Streptomyces* differentiated into dormant uninucleoid spores, which could safeguard genetic information, and resist a wide range of environmental stresses, such as heat, desiccation, and ultraviolet radiation (Bobek et al., 2017; Ma et al., 2020). Notably, some isolates produced diffusible pigments in this study (Supplementary Figure S11), which could be a physiological strategy to cope with the extreme environmental stress (Ma et al., 2020).

*Amycolatopsis*, a rare genus, is widely recognized as a rich source of valuable bioactive natural compounds. Up to now, the secondary metabolites from this genus included polyphenols, linear polyketides, macrolides, macrolactams, peptides, glycoside derivatives, sesquiterpenes, etc. (Banskota et al., 2006; Zhang et al., 2009; Khalil et al., 2017; Pan et al., 2020; Song et al., 2021). Some of them demonstrated significant bioactivities such as antimicrobial, anti-cancer, antioxidant, anti-hyperglycemic, and enzyme inhibition activities (Song et al., 2021). Rifamycins, first isolated from *Amycolatopsis mediterranei* S699 in 1957, are one group of the most representative molecules with bioactivity from *Amycolatopsis* (Sensi, 1957; Sensi et al., 1957, 1959; Song et al., 2021). Hitherto, four rifamycins, namely rifampicin, rifabutin, rifaximin, and rifapentine, derived from the semisynthetic rifamycin SV, are still the principal chemotherapeutic agents for treating tuberculosis, leprosy, pneumococcal meningitis, and infections of human pathogens (Bullock, 1983; Bretonnière et al., 2015; Adams et al., 2017; Greimel et al., 2017). To better investigate and explore secondary metabolites produced by the genera of *Amycolatopsis*, Shi et al. (2019) cultivated the strain of *A. mediterranei* S699 on YMG agar media and isolated 11 members of rifamycins including six new ones. Additionally, 13 rifamycin W congeners with seven new ones including rifamycin W-M1 (**3**), were also isolated from the mutant *A. mediterranei* S699 *Δrif-orf5*, which was constructed by deleting the rif-orf5 gene (Ye et al., 2020).

In this paper, we employed an omics tools including genome sequencing and molecular network-based metabolomics to predict the presence of bioactive secondary metabolites in the strain A133, a Gram-positive rare actinobacterium (Supplementary Figure S12). Firstly, the antiSMASH analysis revealed the biosynthetic potential of strain A133 to produce diverse secondary metabolites including rifamycins. The visualization, clustering, and annotation of the secondary metabolites were achieved by the UPLC-QTOF-MS/MS-based molecular networking (Le Loarer et al., 2023; Zhang et al., 2023). This method enabled us to annotate two families of compounds, namely rifamycins and zampanolides. At least seven rifamycins were annotated in family A. Meanwhile, surprisingly, a unique group of zampanolides were annotated in family B, chemical structure of zampanolide is characterized by a unique 20-membered macrolactone containing a *cis*-2,6-tetrahydropyran, four olenic bonds, three chiral centres, and an uncommon N-acyl hemiaminal side chain with one chiral centre (Chen and Kingston, 2014). To our knowledge, all five zampanolides were isolated from marine sponge (Taufa et al., 2018). Intriguingly, zampanolide was preliminarily annotated from a soil-derived *Amycolatopsis* sp. A133 in this study, which suggested that other types of compounds in this strain are also worthy of further study. Unfortunately, zampanolide was initially predicted, but was not isolated in further isolation process due to the low yield in fermentation broth.

With the assistance of the omics strategy, five rifamycins of family A were isolated from the fermentation broth of the strain A133. These compounds were identified as rifamycin W, protorifamycin I, rifamycin W-M1, proansamycin B, and rifamycin S. Rifamycins, a group of well-known antibiotics of the ansamycin family, are characterized by a 27-membered macrolactam comprising of a naphthalene aromatic group and an aliphatic chain (Wang et al., 2008; Shi et al., 2019). The naphthalene core ends are bridged by an aliphatic chain bearing different chemical moieties (Wang et al., 2008). The structural variations among the five isolated rifamycins mainly concentrated on aliphatic chains and the C-8 position. Specifically, compared to protorifamycin I, proansamycin B differed in the lack of the hydroxyl group at C-34a, whereas rifamycin W exhibited hydroxyl group at C-8. Moreover, the chemical modifications of rifamycin S mainly concentrated on different positions of the aliphatic chain such as C-12, C-25, C-27, C-28, and C-29, including acetylation, methylation, the oxidative cleavage of the C-12/C-29 double bond, etc. Notably, rifamycins with an opened aliphatic chain have ever been isolated from the *Amycolatopsis mediterranei* S699 *∆rif-orf5* and a physical mutagenesis mutant *Nocardia mediterranei* F 1/24 (Ghisalba et al., 1980; Ye et al., 2020), but rifamycin W-M1 was isolated as rifamycin harbouring an opened aliphatic chain from a non-genetically manipulated *Amycolatopsis* strain in this study. Unfortunately, the low yield of the other annotated compounds in family A limited further structural identification in this study.

Protorifamycin I has ever been reported to exhibit the inhibitory activity against *S. aureus* ATCC 25923; while rifamycin W and rifamycin W-M1 did not exhibited antibacterial activities against *S. aureus* ATCC 25923 (Shi et al., 2021); besides, rifamycin S, as an inhibitor of DNA-dependent RNA polymerases, displayed moderate inhibitory actions against *Proteusbacillus vulgaris* CPCC 160013 and *Salmonella enterica Typhimurium* UK-1 χ8956 (Arora and Arjunan, 1992; Shi et al., 2019). In our study, both protorifamycin I and rifamycin S were isolated from the strain A133, which were in consist with results in our antibacterial screening assay.

**Figure 8:**
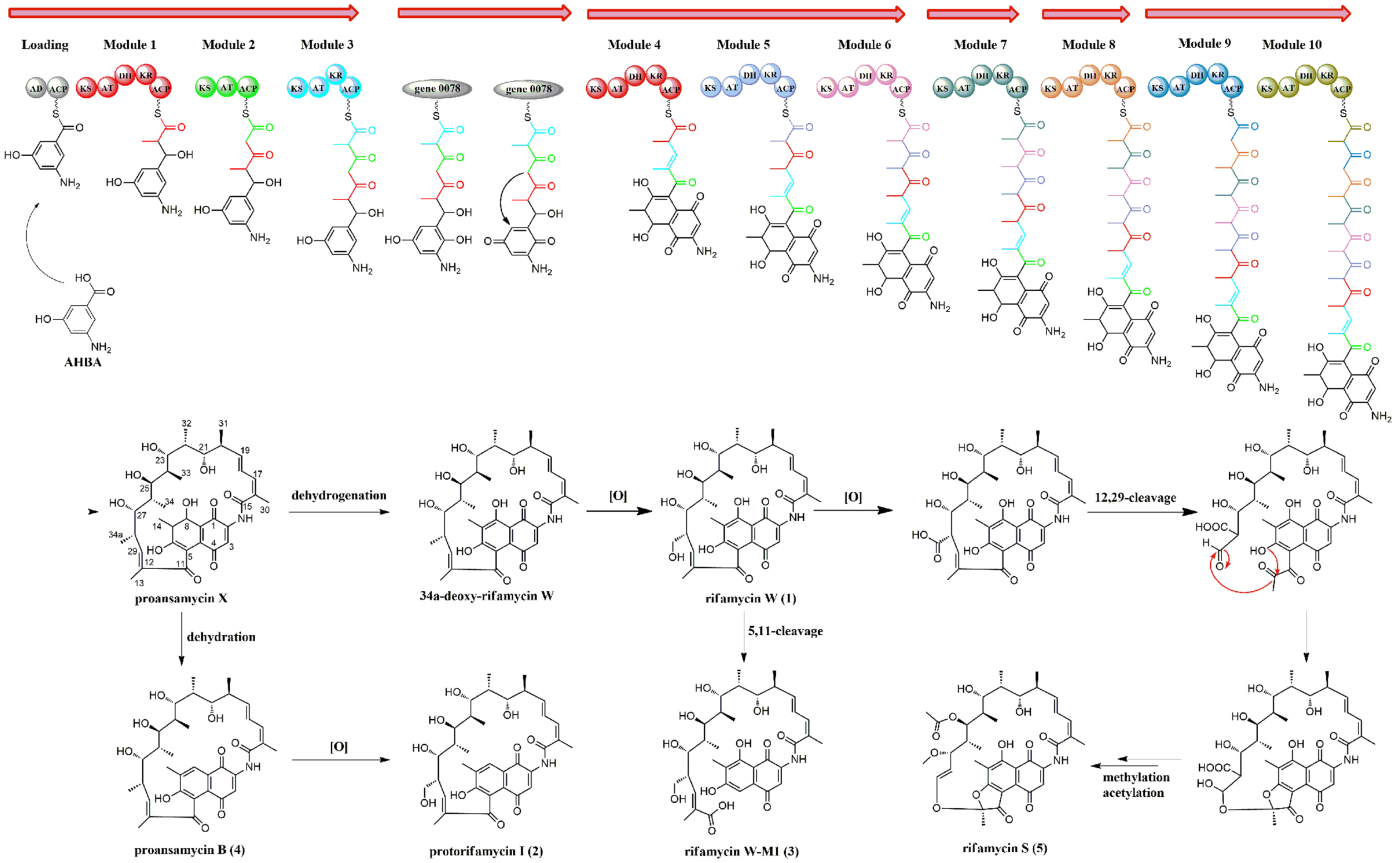
The proposed biosynthetic pathway for rifamycins isolated from *Amycolatopsis* sp. A133.

Biosynthetically, the rifamycins are routinely assembled from the aromatic starter unit 3-amino-5-hydroxybenoic acid (AHBA), and extender units of two malonyl CoA and eight methyl malonyl CoA on a hybrid non-ribosomal peptide synthase/polyketide synthase, followed by the intramolecular amide bond formation to generate proansamycin X. After successive tailoring reactions, the proansamycin X was converted to various rifamycins derivatives. Herein, the cluster 23 is proposed to encode the biosynthesis of rifamycins isolated from the strain A133, which has almost identical gene organization to the known rifamycin cluster within *Amycolatopsis mediterranei* S699 (Supplementary Figure S13). According to the detailed genes annotation and protein Blast analysis, the biosynthesis of rifamycins from the strain A133 is deduced as follow (Figure 8). That is, the genes from locus ctg41_25 to ctg41_29 constitutes the 5 contiguous domains in charge of the undecaketide intermediate generation and the gene 0078 as an extra domain is responsible for the naphthalene structure formation after the first three rounds of extension. Amongst all of the modules, it is the loading module that belongs to an NRPS module, and the other modules are of type I PKS (Xu et al., 2005; Adams et al., 2017). The gene at ctg41_24 encodes an amide synthase, which catalyzes the release of this polyketide chain and intramolecular amide bond formation to generate proansamycin X. Further tailoring reactions of proansamycin X give rise to various rifamycins derivatives. Specifically, the dehydration of C-8, lead to the proansamycin B (2), and subsequent hydroxylation at C-34, give rise to protorifamycin I (4); while, the dehydrogenation of C-8 and the hydroxylation of C-34, lead to the intermediate rifamycin W (1). Furthermore, rifamycin W undergoes C-5/C-11 retro-Claisen cleavage to afford the rifamycin W-M1, which is proposed to be a detoxification mechanism for an over-accumulation of rifamycin W (Shi et al., 2021). While, bioconversion of rifamycin W to rifamycin S (5) is proposed to involve a major polyketide backbone rearrangement, including C-12/C-29 oxidative cleavage, and further methylation and acetylation at C-27, C-25 hydroxyl groups, respectively (Ye et al., 2020; Adams et al., 2017; Shi et al., 2021). In brief, there are so many tailoring enzymes involved in the post-PKS processing, and most of which still remains elusive. Divergent enzymatic mechanisms including substrate spectra, preference, and reaction order will give rise to diversified rifamycin derivatives, which might be reasons for that some nodes remained to be assigned in the molecular network, especially for those grouped with identified rifamycins (**1**–**5**). Besides, the antiSMASH analysis has hinted the extraordinary potential for the strain A133 to produce secondary metabolites, which remains to be further investigated.

## 5. Conclusion

This study investigated the diversity, novelty, and pharmaceutical potential of actinobacterial strains isolated from two soil samples collected on the Western Qinghai-Tibet Plateau. A total of 406 actinobacterial strains were identified and classified into 36 genera in 17 families of 9 orders, which implied the biological diversity of the Western Qinghai-Tibet Plateau as an under-utilized natural ecosystem. Of the 152 strains tested, more than 40% exhibited antagonistic activity against at least one “ESKAPE” pathogen. Notably, seven of these strains demonstrated the ability to inhibit protein translation or induce SOS-response in the “pDualrep2” system. *Amycolatopsis* sp. A133, a rare actinobacterium, was selected as a representative strain for the in-depth chemical investigation. By employing biosynthetic gene cluster mining and GNPS-based metabolomics analysis, five rifamycins were successfully annotated, isolated, and identified. The results illustrate the efficacy of the omics tools in guiding the discovery of bioactive candidates. This paper not only unveils the Western Qinghai-Tibet Plateau as a source of taxonomically diverse and culturable actinobacterial strains with potential for bioactive metabolite production, but also presents a practical approach for efficiently predicting the complex secondary metabolites produced by microorganisms.

## Data availability statement

The datasets presented in this study can be found in online repositories. The names of the repository/repositories and accession number(s) can be found at: https://www.ncbi.nlm.nih.gov/genbank/, OQ569230-OQ569340, https://www.ncbi.nlm.nih.gov/genbank/, OR470596-OR470601, https://www.ncbi.nlm.nih.gov/genbank/, JAVHJU000000000.

## Author contributions

CS conceived the whole study, did the help of the manuscript preparation, supervision, and project administration. LL carried out the experiments and prepared the manuscript. YL helped in editing the manuscript and elucidation of biosynthesis. SL, FL, BZ, and DZ helped in experiments. AN, AI, DL, IO, and PS were responsible for screening the antibacterial mechanisms for samples. All authors contributed to the article and approved the submitted version.

## Footnotes

^1^ https://www.ezbiocloud.net/
