# Supplementary material for: Bioprospecting for the soil-derived actinobacteria and bioactive secondary metabolites on the Western Qinghai-Tibet Plateau
Source: Front Microbiol. 2023 Oct 11;14:1247001. doi: 10.3389/fmicb.2023.1247001 (PMC10599150; doi:10.3389/fmicb.2023.1247001)

Supplementary Material

Bioprospecting for the Soil-Derived Actinobacteria and Bioactive Secondary Metabolites on the Western Qinghai-Tibet Plateau

Lifang Liu[1], Yuyu Liu[1,2], Shaowei Liu[1,2], Arina A. Nikandrova[3,4], Arina N. Imamutdinova[3,5], Dmitrii A. Lukianov[3,5], Ilya A. Osterman[3,5], Petr V. Sergiev[3,5], Benyin Zhang[6], Dejun Zhang[6], Feina Li[7, 8], and Chenghang Sun[1,2,6,9*]

*** Correspondence:**
Chenghang Sun
E-mail: chenghangsun@hotmail.com; sunchenghang@imb.pumc.edu.cn.

Tel.: (00)86-(0)10- 63131856

Content

Table S1. Compositions of the ten selected media for actinobacterial isolation in the study.
Table S2. Taxonomic statistics of the isolated 406 actinobacterial strains.
Table S3. Taxonomic classification of 6 potential new species based on BLAST results of their nearly full-length 16S rRNA gene sequences in the EzBiocloud database.
Table S4. Antibacterial activity of crude samples extracted with ethyl acetate of 63 strains isolated from soils on the Western Qinghai-Tibet Plateau.
Table S5. Putative biosynthetic gene clusters responsible for secondary metabolites in strain A133.
Table S6. Putative compounds obtained from the strain *Amycolatopsis* sp. A133 through the GNPS database and manual dereplication.
Table S7. The characteristics of target compounds **1-5**.
Figure S1. The ^{1}H NMR spectrum of compound 1 in CD_3OD (600 MHz).
Figure S2. The ^{13}C NMR spectrum of compound 1 in CD_3OD (150 MHz).
Figure S3. The ^{1}H NMR spectrum of compound 2 in CD_3OD (600 MHz).
Figure S4. The ^{13}C NMR spectrum of compound 2 in CD_3OD (150 MHz).
Figure S5. The ^{1}H NMR spectrum of compound 3 in CD_3OD (600 MHz).
Figure S6. The ^{13}C NMR spectrum of compound 3 in CD_3OD (150 MHz).
Figure S7. The ^{1}H NMR spectrum of compound 4 in CD_3OD (600 MHz).
Figure S8. The ^{13}C NMR spectrum of compound 4 in CD_3OD (150 MHz).
Figure S9. The ^{1}H NMR spectrum of compound 5 in CD_3OD (600 MHz).
Figure S10. The ^{13}C NMR spectrum of compound 5 in CD_3OD (150 MHz).
Figure S11. Pictures of representative diffusible pigments produced by actinobacteria in the study.
Figure S12. The photo graphs of Amycolatopsis sp. A133 on the plate and picture after Gram-staining. (**A**) The photo graph of *Amycolatopsis* sp. A133 on the plate. (**B**) The picture after Gram-staining of *Amycolatopsis* sp. A133.
Figure S13. Comparison of the putative biosynthetic gene cluster from strain A133 with the reported rifamycin cluster (BGC0000316).

Table S1. Compositions of the ten selected media for actinobacterial isolation in the study.

NO.	Name	Composition (In 1.0 L distilled water)
M1	R_2A medium	R_2A (3.2 g), Agar (20.0 g), pH 7.2–7.5
M2	Modified proline medium	Proline (5.00 g), Agar (20.0 g), pH 7.2–7.5
M3	Glyceryl-arginine medium	Glycerol (12.50 g), Arginine (2.00 g), $K_2HPO_4 \cdot 3H_2O$ (2.0 g), $MgSO_4 \cdot 7H_2O$ (0.05 g), $FeSO_4 \cdot 7H_2O$ (0.01 g), $CuSO_4 \cdot 5H_2O$ (0.001 g), $ZnSO_4 \cdot 7H_2O$ (0.001 g), $MnSO_4 \cdot H_2O$ (0.001 g), Agar (20.0 g), pH 9.0–10.0
M4	Modified ISP 2 medium	Yeast extract (5.0 g), Glucose (4.0 g), Malt extract (4.0 g), Vitamin mixture [a] (1.0 mL), Trace salt [b] (1.0 mL), Agar (20.0 g), pH 7.2–7.5
M5	Raffinose-histidine medium	Raffinose (1.0 g), Histidine (0.1 g), KCl (1.7 g), $Na_2HPO_4 \cdot 12H_2O$ (0.5 g), $MgSO_4 \cdot 7H_2O$ (0.05 g), $FeSO_4 \cdot 7H_2O$ (0.1 g), $CaCO_3$ (0.02 g), Vitamin mixture [a] (1.0 mL), Agar (20.0 g), pH 7.2–7.5
M6	CMKA medium	Casein acids hydrolysate (0.5 g), Mannitol (1.5 g), KNO_3 (1.0 g), $(NH4)_2SO_4$ (2.0 g), $K_2HPO_4 \cdot 3H_2O$ (0.5 g), $CaCO_3$ (0.5 g), NaCl (10.0 g), KCl (5.0 g), $MgCl_2$ (1.0 g), Agar (20.0 g), pH 7.2–7.5
M7	Glycerol-casein medium	Glycerol (10.0 g), Casein (0.3 g), KNO_3 (2.0 g), NaCl (2.0 g), $K_2HPO_4 \cdot 7H_2O$ (2.0 g), $MgSO_4 \cdot 7H_2O$ (0.05 g), $FeSO_4 \cdot 7H_2O$ (0.01 g), $CaCO_3$ (0.2 g), Agar (20.0 g), pH 7.2–7.5
M8	Modified HV medium	Soluble starch (2.0 g), KNO_3 (0.5 g), KCl (1.7 g), $MgSO_4 \cdot 7H_2O$ (0.5 g), $Na_2HPO_4 \cdot 12H_2O$ (0.5 g), NaCl (0.5 g), $CaCO_3$ (0.02 g), $FeSO_4 \cdot 7H_2O$ (0.01 g), Vitamin mixture [a] (1.0 mL), Agar (20.0 g), pH 7.2–7.5
M9	Sodium propionate-casein medium	Sodium propionate (2.0 g), Casein hydrolysate (0.3 g), KNO_3 (0.1 g), Agar (20.0 g), pH 7.2-7.5
M10	Humic acid medium	Humic acid (0.5 g), $Na_2HPO_4 \cdot 12H_2O$ (0.25 g), $MgSO_4 \cdot 7H_2O$ (0.025 g), $FeSO_4 \cdot 7H_2O$ (0.005 g), $CaCO_3$ (0.01 g), Vitamin B1 (0.002 g), Agar (20.0 g), pH 7.2–7.5

Note: [a]Vitamin mixture: thiamine (0.1 g), pyridoxine (0.1 g), riboflavin (0.1 g), niacin (0.1 g), biotin (0.1 g), distilled water (100.0 mL). [b]Trace salt solution: $FeSO_4 \cdot 7H_2O$ (0.2 g), $MnCl_2 \cdot 4H_2O$ (0.01 g), $ZnSO_4 \cdot 7H_2O$ (0.01 g), distilled water (100.0 mL).

Table S2. Taxonomic statistics of the isolated 406 actinobacterial strains.

Taxon			No. of isolates
**Order (9)**	**Family (17)**	**Genus (36)**	
Streptomycineae	*Streptomycetaceae*	*Streptomyces*	269
Micromonosporales	*Micromonosporaceae*	*Micromonospora*	2
Mycobacteriales	*Nocardiaceae*	*Rhodococcus*	4
		Nocardia	5
	Mycobacteriaceae	*Mycolicibacterium*	1
		Mycobacterium	2
	Gordoniaceae	*Williamsia*	1
Streptosporangiales	*Streptosporangiaceae*	*Nonomuraea*	1
Propionibacteriales	*Nocardioidaceae*	*Aeromicrobium*	3
	Kribbellaceae	*Kribbella*	7
	Propionibacteriaceae	*Auraticoccus*	1
		Microlunatus	2
Pseudonocardiales	*Pseudonocardiaceae*	*Saccharothrix*	2
		Lentzea	1
		Amycolatopsis	7
		Pseudonocardia	2
		Saccharopolyspora	1
		Umezawaea	1
Geodermatophilales	*Geodermatophilaceae*	*Modestobacter*	1
Kineosporiale	*Kineosporiaceae*	*Kineococcus*	1
Micrococcales	*Microbacteriaceae*	*Microbacterium*	44
		Agrococcus	8
		Leifsonia	1
		Curtobacterium	3
		Rathayibacter	2
		Mycetocola	2
		Labedella	3
		Planctomonas	1
		Diaminobutyricimonas	1
	Bogoriellaceae	*Georgenia*	1
	Cellulomonadaceae	*Cellulomonas*	2
	Promicromonosporaceae	*Promicromonospora*	2
	Micrococcaceae	*Kocuria*	4
		Micrococcus	2
		Arthrobacter	13
		Paenarthrobacter	3
Total number of actinobacteria isolated			406

Table S3. Taxonomic classification of 6 potential new species based on BLAST results of their nearly full-length 16S rRNA gene sequences in the EzBiocloud database.

NO.	Strain NO.	GenBank Accession NO.	Length of blasted 16S rRNA gene sequences (bp)	Top-hit taxon	Similarity （%）	Family assignment
1	G108-PJ	OR470600	1392	*Galbitalea soli* KIS82-1^T	97.99	*Microbacteriaceae*
2	B415-PJ	OR470597	1386	*Microbacterium keratanolyticum* IFO 13309^T	98.41	*Microbacteriaceae*
3	E472-PJ	OR470599	1393	*Nonomuraea dietziae* DSM 44320^T	98.64	*Streptosporangiaceae*
4	B415-1-PJ	OR470598	1401	*Streptomyces badius* NRRL B-2567^T	97.91	*Streptomycetaceae*
5	B663-PJ	OR470596	1388	*Streptomyces nymphaeiformis* SFB5A^T	98.63	*Streptomycetaceae*
6	A150-3-1-PJ	OR470601	1388	*Streptomyces nymphaeiformis* SFB5A^T	98.63	*Streptomycetaceae*

Table S4. Antibacterial activity of crude samples extracted with ethyl acetate of 63 strains isolated from soils on the Western Qinghai-Tibet Plateau.

NO.	Isolates (GenBank accession NO.)	Top-hit taxon (Pairwise similarity)	Activity [a]											
			E. coli		*P. aeruginosa*		*K. pneumoniae*		*A. baumanni*		*S. aureus*		*E. faecalis*	
			S	R	S	R	S	R	S	R	S	R	S	R
1	A404 (OQ569266)	*Streptomyces phaeoluteigriseus* DSM 41896^T, 100%	-	-	-	-	10	-	10	-	14	13	-	-
2	D803 (OQ569267)	*Streptomyces avidinii* NBRC 13429^T, 99.88%	-	-	-	-	10	-	16	10	22	18	14	-
3	A437-3 (OQ569270)	*Streptomyces xanthophaeus* NRRL B-5414^T, 99.87%	-	-	-	-	-	-	-	-	13	12	10	-
4	A809 (OQ569271)	*Streptomyces humidus NBRC* 12877^T, 99.88%	-	-	-	-	-	-	-	-	11	-	-	-
5	A820 (OQ569272)	*Streptomyces turgidiscabies* ATCC 700248^T, 100%	-	-	-	-	-	-	11	12	19	11	14	-
6	H130-1 (OQ569274)	*Streptomyces purpureus* NBRC 13927T, 99.74%	-	-	-	-	-	-	-	12	12	20	-	-
7	A603 (OQ569276)	*Streptomyces purpureus* NBRC 13927^T, 99%	15	10	-	-	17	13	21	21	40	29	28	-

8	C1006-1 (OQ569277)	*Streptomyces fumanus* NBRC 13042T, 99.48%	-	-	-	-	-	-	-	-	10	14	-	-
9	E404-1-2 (OQ569278)	*Streptomyces nigrescens* NBRC 12894T, 99.74%	-	-	-	-	13	-	15	14	22	14	12	-
10	E404-2 (OQ569279)	*Streptomyces nigrescens* NBRC 12894^T, 99.74%	10	-	10	-	18	-	14	18	22	15	-	-
11	B614 (OQ569280)	*Streptomyces lavendulae subsp. Lavendulae* NRRL B-2774^T, 100%	13	-	-	-	-	10	-	-	-	10	-	-
12	A601 (OQ569281)	*Streptomyces badius* NRRL B-2567^T, 100%	-	-	-	-	10	-	-	-	14	12	15	-
13	D106-2 (OQ569282)	*Streptomyces spinoverrucosus* NBRC 14228^T, 99.74%	-	-	-	-	-	-	-	-	-	14	-	-
14	H829-1-1 (OQ569283)	*Streptomyces durmitorensis* MS405^T, 100%	-	-	-	-	11	12	10	10	12	11	-	-
15	E7101 (OQ569284)	*Streptomyces kurssanovii* NBRC 13192^T, 99.87%	-	-	-	-	-	-	-	-	-	-	-	12
16	A609 (OQ569285)	*Streptomyces polyantibioti*	-	-	-	-	14	-	12	10	16	12	16	-

		cus SPR^{T}, 99.21%												
17	A822 (OQ569286)	*Streptomyces avermitilis* MA-4680^{T}, 100%	10	-	-	-	-	-	-	-	-	10	-	-
18	D816-1 (OQ569287)	*Streptomyces eurythermus* ATCC 14975^{T}, 99.21%	-	10	-	-	10	-	10	10	29	21	22	-
19	H1002-1 (OQ569288)	*Streptomyces narbonensis* NBRC 12801^{T}, 99.74%	-	-	-	-	-	-	13	12	10	-	-	-
20	A712 (OQ569290)	*Streptomyces cirratus* NRRL B-3250^{T}, 99.74%	-	-	-	-	10	-	-	10	21	22	14	10
21	E1003 (OQ569292)	*Streptomyces bacillaris* NBRC 13487^{T}, 99.74%	-	-	-	-	-	-	10	-	14	-	-	-
22	A923 (OQ569293)	*Streptomyces bacillaris* NBRC 13487^{T}, 99.74%	-	-	-	-	-	10	15	14	27	21	17	10
23	B712 (OQ569294)	*Streptomyces setonii* NRRL ISP-5395^{T}, 99.48%	-	-	-	-	-	-	11	12	20	11	-	10
24	H121-2 (OQ569295)	*Streptomyces lienomycini* ATCC 43687^{T}, 99.87%	-	-	-	-	-	-	-	-	13	10	-	-
25	A442-2 (OQ569296)	*Streptomyces griseoruber*	-	-	-	-	-	-	-	-	15	14	-	-

		NRRL B-1818T, 99.35%												
26	A-F813-1 (OQ569297)	*Streptomyces griseoruber* NRRL B-1818^{T}, 99.61%	-	-	-	-	-	-	14	13	19	15	-	-
27	F1021-1 (OQ569298)	*Streptomyces cyaneus* NRRL B-2296^{T}, 99.87%	10	10	-	-	12	-	11	12	20	15	11	10
28	E612-4 (OQ569299)	*Streptomyces huasconensis* HST28^{T}, 100%	-	-	-	-	-	-	-	-	13	11	-	-
29	A806 (OQ569300)	*Streptomyces ederensis* NBRC 15410^{T}, 99.6%	12	-	-	-	10	-	16	14	26	20	23	-
30	E702 (OQ569301)	*Streptomyces arboris* T258^{T}, 99.34%	-	-	-	-	-	-	11	11	13	-	-	-
31	E1002 (OQ569302)	*Streptomyces lateritius* LMG 19372^{T}, 100%	-	-	-	-	-	-	11	12	-	-	-	-
32	A608 (OQ569303)	*Streptomyces glomeroaurantiacus* NBRC 15418^{T}, 100%	-	-	-	-	-	-	-	-	20	-	-	-
33	D705 (OQ569304)	*Streptomyces flavovirens* NBRC 3716^{T}, 100%	-	-	-	-	-	-	-	-	18	14	16	-
34	D706-1 (OQ569305)	*Streptomyces gobitricini* NBRC	-	-	-	-	-	-	-	-	19	-	12	-

		15419^T, 98.69%												
35	E118 (OQ569308)	*Streptomyces tauricus* JCM 4837^T, 100%	-	-	-	-	-	-	-	-	10	-	-	-
36	H805-1 (OQ569310)	*Streptomyces zaomyceticus* NBRC 13348^T, 99.74%	-	10	-	-	18	-	22	20	38	26	22	-
37	B407A (OQ569311)	*Streptomyces xanthochromogenes* NRRL B-5410^T, 98.62%	-	-	-	-	-	-	-	-	10	-	-	-
38	E113 (OQ569312)	*Streptomyces rectiviolaceus* NRRL B-16374^T, 100%	-	-	-	-	-	-	-	-	13	-	-	-
39	A706 (OQ569313)	*Streptomyces dioscori* A217^T, 99.61%	12	10	-	-	15	10	17	16	26	22	23	-
40	H802-1 (OQ569315)	*Streptomyces spiroverticillatus* NBRC 12821^T, 99.63%	-	-	-	-	-	-	-	-	14	10	-	-
41	E728 (OQ569316)	*Streptomyces nitrosporeus* NBRC 3362^T, 99.75%	-	-	-	-	-	-	-	11	14	11	-	-
42	A821 (OQ569320)	*Streptomyces olivochromogenes* DSM 40451^T, 100%	10	10	-	-	-	-	-	10	10	-	-	-

43	A462 (OQ569321)	*Streptomyces microflavus* DSM 40593^{T}, 100%	-	10	-	-	-	-	-	-	-	-	-	-
44	E720 (OQ569322)	*Streptomyces levis* NBRC 15423^{T}, 99.74%	10	-	-	-	10	-	13	11	14	14	14	-
45	A911-1 (OQ569324)	*Streptomyces rishiriensis* NBRC 13407^{T}, 100%	-	-	-	-	-	-	-	-	11	10	-	-
46	A616-1 (OQ569326)	*Streptomyces maoxianensis* NEAU-Spg16^{T}, 99.74%	10	-	-	-	11	-	-	-	15	15	-	11
47	A147 (OQ569327)	*Streptomyces resistomycificus* NRRL ISP-5133^{T}, 99.87%	10	10	-	-	-	-	-	-	-	-	-	-
48	D830-2 (OQ569231)	*Promicromonospora alba* 1C-HV12^{T}, 99.74%	-	-	-	-	-	-	-	14	-	-	-	-
49	H834-1 (OQ569232)	*Nonomuraea dietziae* DSM 44320^{T}, 100%	-	-	-	-	-	-	-	-	-	-	10	12
50	F824 (OQ569329)	*Nonomuraea dietziae* DSM 44320^{T}, 98.5%	-	-	-	-	-	-	-	-	10	-	-	-
51	B665 (OQ569330)	*Aeromicrobium massiliense* JC14^{T}, 98.69%	-	-	-	-	-	-	-	10	-	-	-	-

52	B812-2 (OQ569233)	*Aeromicrobium choanae* 9H-4^T, 100%	-	-	-	-	-	-	-	-	11	10	10	-
53	F147-1 (OQ569234)	*Saccharopolyspora flava* NBRC 16345^T, 99.74%	-	-	-	-	-	-	-	-	10		-	-
54	E650 (OQ569332)	*Amycolatopsis orientalis* DSM 40040^T, 100%	-	-	-	-	-	-	-	-	18	15	14	-
55	A129 (OQ569333)	*Amycolatopsis pretoriensis* DSM 44654T, 98.95%	-	-	-	-	-	-	-	-	16	22	12	-
56	A133 (OQ569235)	*Amycolatopsis pretoriensis* DSM 44654^T, 98.82%	-	-	-	-	-	11	-	-	27	26	16	-
57	A738 (OQ569335)	*Amycolatopsis balhimycina* FH 1894^T, 98.81%	-	-	-	-	-	-	-	-	15	11	10	-
58	F1022-2 (OQ569336)	*Kribbella albertanoniae* BC640^T, 100%	-	-	-	-	-	12	-	11	-	-	-	-
59	A834 (OQ569236)	*Kribbella flavida* DSM 17836^T, 99.21%	-	-	-	-	-	12	-	10	10	-	-	-
60	D714-3-1 (OQ569338)	*Kribbella ginsengisoli Gsoil* 001^T, 98.69%	-	-	-	-	-	-	12	13	12	10	13	-
61	D831 (OQ569237)	*Nocardia tenerifensis* NBRC	-	-	-	-	10	-	-	-	15	14	10	-

		101015^T, 99.75%												
62	A104 (OQ569242)	*Paenarthrobacter nitroguajacolicus* G2-1^T, 100%	-	-	-	-	-	-	-	-	-	11	-	-
63	A102 (OQ569340)	*Paenarthrobacter nicotinovorans* DSM 420^T, 99.87%	-	-	-	-	-	-	-	-	-	10	-	-

[a]The diameters of the inhibition zones: mm; -, no inhibitory activity.

Table S5. Putative biosynthetic gene clusters responsible for secondary metabolites in strain A133[a].

Cluster	Type	From	To	Most similar known cluster	Type	Similarity
Cluster 1	Terpene	315578	336504	Isorenieratene	Terpene	42 %
Cluster 2	NRPS-like, NRPS, T1PKS	283399	342607	Crochelin A	NRP + Polyketide	16 %
Cluster 3	NRPS	116023	194514	Cyclofaulknamycin	Polyketide	12 %
Cluster 4	NI-siderophore	470520	482250	Nonactin, Monactin, Dinactin, Trinactin, Tetranactin	Polyketide	33 %
Cluster 5	NRPS, T2PKS	573605	687175	Arixanthomycins	Polyketide	57 %
Cluster 6	NRPS	20945	71404	Desertomycins	NRP	5 %
Cluster 7	T1PKS	5961	47492	–	–	–
Cluster 8	HglE-KS, T1PKS	192389	243658	Hexacosalactone A	Other	11 %
Cluster 9	Redox-cofactor	8787	30794	Lankacidin C	NRP + Polyketide	20 %
Cluster 10	HglE-KS	194114	243673	Hexacosalactone A	Other	11 %
Cluster 11	T1PKS	27719	74126	–	–	–
Cluster 12	Terpene	194553	205793	2-methylisoborneol	Terpene	50 %
Cluster 13	Lanthipeptide-class-III	66488	89064	–	–	–
Cluster 14	Ectoine	9869	20261	Ectoine	Other	100 %

Cluster 15	NRPS	39457	102845	Scabichelin	NRP	80 %
Cluster 16	RiPP-like	5466	17787	Granaticin	Polyketide	5 %
Cluster 17	NRPS	21501	78265	Amycolamycins	Polyketide	45 %
Cluster 18	Ectoine	86196	96567	Kosinostatin	NRP + Polyketide	10 %
Cluster 19	NRPS, T1PKS	136475	292770	α-lipomycin	NRP	27 %
Cluster 20	HglE-KS	128280	171378	–	–	–
Cluster 21	NRPS	30414	175804	Cyclomain D	NRP	17 %
Cluster 22	Terpene, T1PKS, others	206871	260586	Symocyclinone D8	Saccharide + Polyketide	18 %
Cluster 23	T1PKS, NRPS-like	5159	95609	Rifamorpholines	Polyketide	73 %
Cluster 24	Indole	14384	35484	Fortimicin	Saccharide	4 %
Cluster 25	NRPS, T1PKS	189950	307617	Detoxins	NRP + Polyketide	100 %
Cluster 26	NRPS	357819	457395	Colibrimycin	NRP + Other	19 %
Cluster 27	RiPP-like	127541	139373	GP6738	NRP	9 %
Cluster 28	Lanthipeptide-class-v	144680	200347	Phenalinolactone A	Terpene + Saccharide	5 %

Cluster 29	Butyrolactone	2463	13266	–	–	–
Cluster 30	RiPP-like	207145	219031	Notonesomycin A	Other	3 %
Cluster 31	Terpene	173656	195833	Geosmin	Terpene	100 %
Cluster 32	T1PKS	1	27958	Iasalocid	Polyketide	7 %
Cluster 33	Redox-cofactor	94570	116641	Lankacidin C	NRP + Polyketide	13 %
Cluster 34	T1PKS	152493	199500	ECO-0501	Polyketide	4 %

[a] Secondary metabolite types detected by antiSMASH: T1PKS, type I PKS cluster; T2PKS, type II PKS cluster; NRPS, nonribosomal peptide synthetase cluster; other cluster containing a secondary metabolite, related protein that did not fit into any other category. PKS cluster. The similarity represents the percentage of homologous genes in the query cluster that are present in the hit cluster of the antiSMASH database. –, not available

Table S6. Putative compounds obtained from the bioactive component of strain *Amycolatopsis* sp. A133 through the GNPS database and manual dereplication.

Putative compound	Molecular formula	Chemical structure	m/z (detected)	Protonated adducts	t_R/s
Rifamycin W	$C_{35}H_{45}NO_{11}$	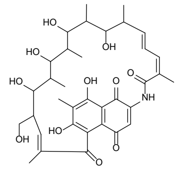	656.307	$[M + H]^+$	481.65
Protorifamycin I	$C_{35}H_{45}NO_{10}$	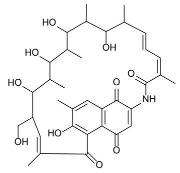	640.310	$[M + H]^+$	445.21
Rifamycin W-M1	$C_{35}H_{47}NO_{12}$	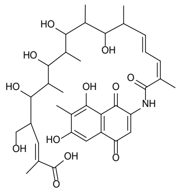	674.316	$[M + H]^+$	560.60
Proansamycin B	$C_{35}H_{45}NO_{9}$	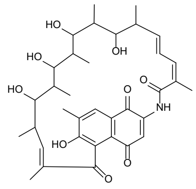	624.314	$[M + H]^+$	518.73
Rifamycin S	$C_{37}H_{45}NO_{12}$	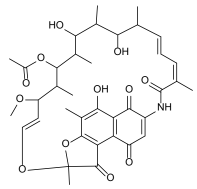	696.309	$[M + H]^+$	658.09
Rifamycin W-hemiacetal	$C_{35}H_{43}NO_{11}$	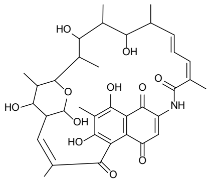	654.291	$[M + H]^+$	541.99

20-hydroxyrifamycin S	$C_{37}H_{45}NO_{13}$	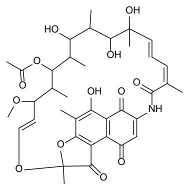	712.305	$[M + H]^+$	565.98
Zampanolide	$C_{29}H_{37}NO_6$	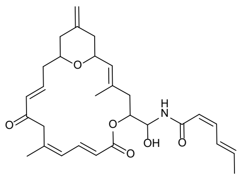	496.271	$[M + H]^+$	1061.59

Table S7. The characteristics of target compounds **1-5**.

Characteristic data of rifamycin W (**1**)

^{1}H NMR (600 MHz, CD_3OD): δ 7.47 (s, 1H), 6.52 (dd, J = 16.0, 10.8 Hz, 1H), 6.46 (d, J = 9.4 Hz, 1H), 6.24 (d, J = 10.8 Hz, 1H), 6.08 (dd, J = 16.0, 6.8 Hz, 1H), 4.34 (t, J = 1.7 Hz, 1H), 4.03 (d, J = 9.7 Hz, 1H), 4.00 (dd, J = 10.2, 1.6 Hz, 1H), 3.60 (dd, J = 11.0, 7.1 Hz, 1H), 3.51–3.48 (m, 1H), 3.46 (dd, J = 10.5, 2.6 Hz, 1H), 2.65–2.62 (m, 1H), 2.38–2.34 (m, 1H),, 2.08 (s, 3H), 2.08 (s, 3H), 2.05 (s, 3H), 1.89–1.82 (m, 1H), 1.84–1.78 (m, 1H), 1.42–1.36 (m, 1H), 1.05 (d, J = 7.1 Hz, 3H), 0.91 (d, J = 7.0 Hz, 3H), 0.71 (d, J = 6.8 Hz, 3H), 0.43 (d, J = 7.0 Hz, 3H);

^{13}C NMR (150 MHz, CD_3OD): δ 202.53, 187.90, 180.77, 171.97, 164.40, 143.04, 141.52, 140.75, 140.56, 135.20, 132.03, 129.50, 128.84, 126.33, 118.32, 117.03, 105.28, 78.93, 74.92, 71.11, 69.68, 64.98, 49.58, 44.04, 39.03, 37.86, 34.27, 22.06, 20.10, 18.02, 12.72, 11.65, 11.10, 8.82, 8.43;

HRESIMS: m/z 656.3074 [M + H]$^{+}$ (calcd. for 656.3071), MW, $C_{35}H_{45}NO_{11}$.

Characteristic data of protorifamycin I (**2**)

^{1}H NMR (600 MHz, CD_3OD): δ 7.96 (s, 1H), 7.61 (s, 1H), 6.48 (dd, J = 15.9, 10.8 Hz, 1H), 6.33 (d, J = 9.3 Hz, 1H), 6.24 (d, J = 10.8 Hz, 1H), 6.06 (dd, J = 15.9, 6.8 Hz, 1H), 4.39 (d, J = 1.5 Hz, 1H), 4.03 (dd, J = 9.7, 2.1 Hz, 1H), 3.99 (dd, J = 10.2, 1.7 Hz, 1H), 3.58 (dd, J = 10.9, 8.1 Hz, 1H), 3.47 (dd, J = 10.2, 2.4 Hz, 1H), 3.42 (dd, J = 10.9, 6.4 Hz, 1H), 2.67–2.61 (m, 1H), 2.36 (s, 3H), 2.35–2.32 (m, 1H), 2.10 (s, 3H), 2.08 (s, 3H), 1.86–1.82 (m, 1H), 1.82–1.75 (m, 1H), 1.44–1.38 (m, 1H), 1.05 (d, J = 7.0 Hz, 3H), 0.91 (d, J = 6.9 Hz, 3H), 0.72 (d, J = 6.8 Hz, 3H), 0.40 (d, J = 7.0 Hz, 3H);

^{13}C NMR (150 MHz, CD_3OD): δ 200.69, 187.40, 180.30, 172.23, 159.42, 141.95, 141.75, 141.35, 140.91, 134.94, 133.20, 132.13, 131.61, 131.50, 128.67, 126.15, 123.77, 118.20, 78.92, 74.70, 71.11, 68.83, 64.33, 49.49, 44.02, 39.10, 37.88, 34.29, 20.15, 18.01, 17.03, 12.52, 11.67, 11.14, 8.85;

HRESIMS: m/z 640.3118 [M + H]$^{+}$ (calcd. for 640.3122), MW, $C_{35}H_{45}NO_{10}$.

Characteristic data of rifamycin W-M1 (**3**)

^{1}H NMR (600 MHz, CD_3OD): δ 7.58 (s, 1H), 7.02 (s, 1H), 6.89 (d, J = 10.1 Hz, 1H), 6.83 (dd, J = 15.1, 11.1 Hz, 1H), 6.53 (d, J = 11.1 Hz, 1H), 6.04 (dd, J = 15.1, 8.1 Hz, 1H), 4.12 (d, J = 4.5 Hz, 1H), 4.03 (d, J = 9.8 Hz, 1H), 3.82 (d, J = 8.9 Hz, 1H), 3.63 (m, 1H), 3.60–3.54 (m, 1H), 3.49 (m, 1H), 2.82–2.77 (m, 1H), 2.47–2.43 (m, 1H), 2.08 (s, 3H), 2.06 (s, 3H), 1.93–1.89 (m, 1H), 1.88 (s, 3H), 1.85–1.71 (m, 2H), 1.00 (d, J = 6.8 Hz, 3H), 0.95 (d, J = 7.0 Hz, 3H), 0.89 (d, J = 7.0 Hz, 3H), 0.81 (d, J = 7.0 Hz, 3H);

^{13}C NMR (150 MHz, CD_3OD): δ 186.41, 183.60, 172.1, 169.74, 165.20, 163.75, 146.51, 142.90, 142.27, 139.02, 132.14, 131.18, 129.02, 127.52, 118.10, 116.89, 108.77, 108.12, 79.02, 75.61, 73.00, 71.92, 63.88, 46.88, 42.35, 40.68, 37.73, 36.34, 20.57, 17.37, 13.5, 10.61, 10.55, 10.38, 8.01;

HRESIMS: m/z 674.3159 $[M + H]^+$ (calcd. for 674.3177), MW, $C_{35}H_{47}NO_{12}$.

Characteristic data of proansamycin B (**4**)

^1H NMR (600 MHz, CD_3OD): δ 7.95 (s, 1H), 7.60 (s, 1H), 6.49 (dd, J = 15.9, 10.9 Hz, 1H), 6.43 (d, J = 9.2 Hz, 1H), 6.24 (d, J = 10.9 Hz, 1H), 6.07 (dd, J = 15.9, 6.8 Hz, 1H), 4.04 – 3.99 (m, 2H), 3.97 (d, J = 10.1 Hz, 1H), 3.47 (dd, J = 10.1, 2.5 Hz, 1H), 2.60 (m, 1H), 2.36 (s, 3H), 2.36–2.30 (m, 1H), 2.08 (s, 3H), 2.05 (s, 3H), 1.87–1.85 (m, 1H), 1.82–1.73 (m, 1H), 1.45–1.41 (m, 1H), 1.07 (d, J = 7.1 Hz, 3H), 1.05 (d, J = 7.1 Hz, 3H), 0.92 (d, J = 6.9 Hz, 3H), 0.72 (d, J = 6.8 Hz, 3H), 0.37 (d, J = 7.0 Hz, 3H);

^{13}C NMR (150 MHz, CD_3OD): δ 199.92, 187.38, 180.23, 174.89, 172.17, 160.49, 146.52, 141.90, 141.41, 138.10, 134.95, 132.10, 131.62, 131.52, 128.60, 126.19, 118.22, 78.85, 74.79, 74.07, 71.26, 43.93, 41.01, 39.17, 37.91, 34.39, 20.15, 19.35, 18.04, 17.06, 11.82, 11.27, 11.18, 8.83;

HRESIMS: m/z 624.3193 $[M + H]^+$ (calcd. for 624.3186), MW, $C_{35}H_{45}NO_9$.

Characteristic data of rifamycin S (**5**)

^1H NMR (600 MHz, CD_3OD): δ 7.62 (s, 1H), 6.26 (s, 1H), 6.22 (dd, J = 14.5, 10.6 Hz, 1H), 6.17 (d, J = 12.4 Hz, 1H), 5.88 (dd, J = 14.5, 6.6 Hz, 1H), 5.25 (dd, J = 12.4, 7.8 Hz, 1H), 4.92 (d, J = 10.6 Hz, 1H), 3.67 (d, J = 9.7 Hz, 1H), 3.35 (dd, J = 7.9, 2.9 Hz, 1H), 3.04 (s, 3H), 3.06–3.04 (m, 1H), 2.26 (s, 3H), 2.34–2.27 (m, 1H), 2.00 (s, 3H), 1.91 (s, 3H), 1.82–1.73 (m, 2H), 1.65 (s, 3H), 1.51–1.47 (m, 1H), 0.96 (d, J = 7.0 Hz, 3H), 0.83 (d, J = 6.9 Hz, 3H), 0.66 (d, J = 6.9 Hz, 3H), 0.07 (d, J = 7.1 Hz, 3H);

^{13}C NMR (150 MHz, CD_3OD): δ 194.05, 189.03, 184.33, 174.32, 172.91, 172.01, 168.62, 145.59, 142.19, 141.37, 134.31, 132.34, 125.49, 118.19, 118.00, 117.07, 109.48, 82.24, 77.94, 74.54, 73.94, 56.92, 40.05, 38.89, 38.73, 34.03, 22.23, 21.10, 20.18, 17.52, 11.94, 11.43, 9.28, 7.81;

HRESIMS: m/z 696.3040 $[M + H]^+$ (calcd. For 696.3020), MW, $C_{37}H_{45}NO_{12}$.

Figure S1. The ^{1}H NMR spectrum of compound **1** in CD_3OD (600 MHz).

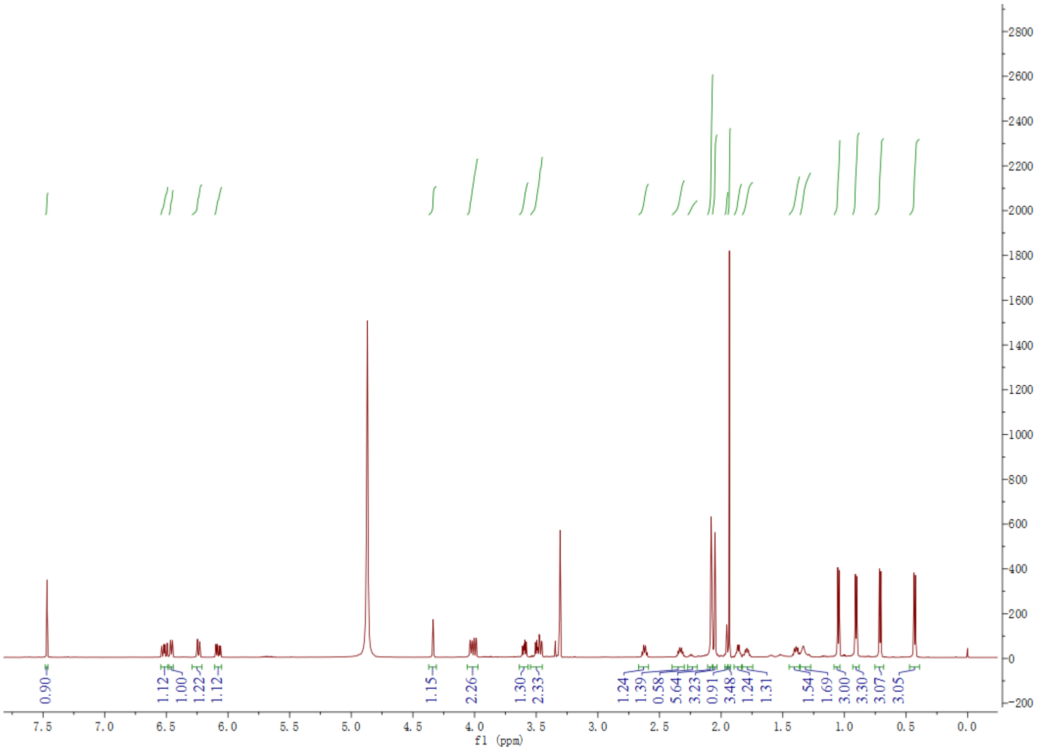

Figure S2. The ^{13}C NMR spectrum of compound **1** in CD_3OD (150 MHz).

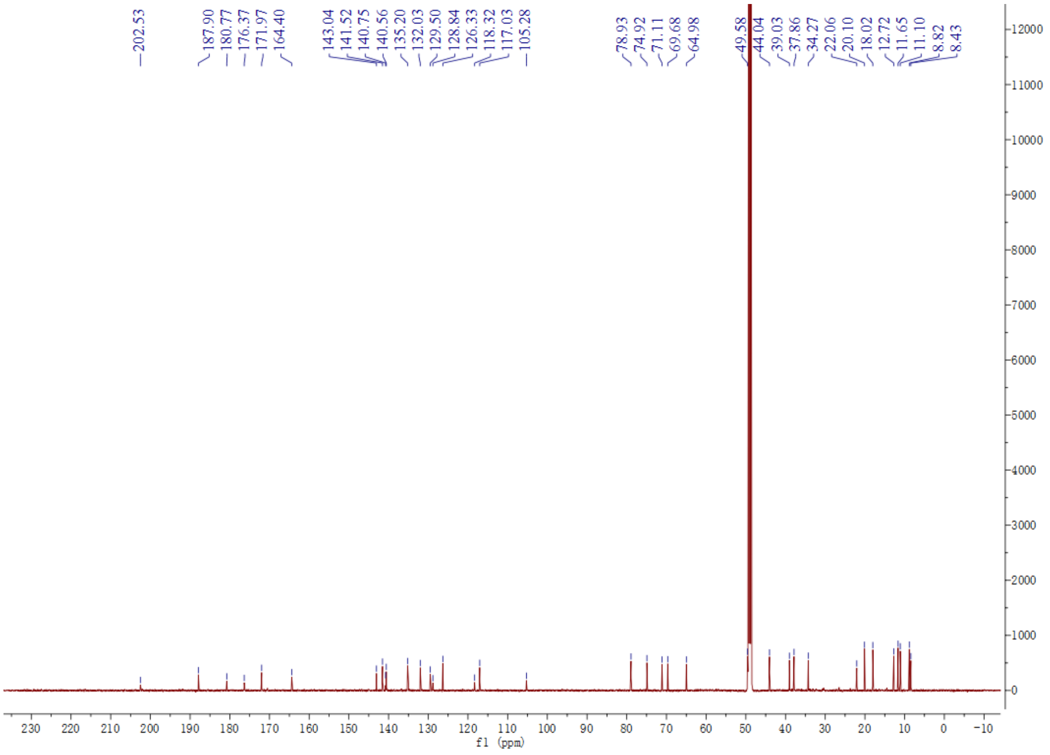

Figure S3. The ^{1}H NMR spectrum of compound **2** in CD_3OD (600 MHz).

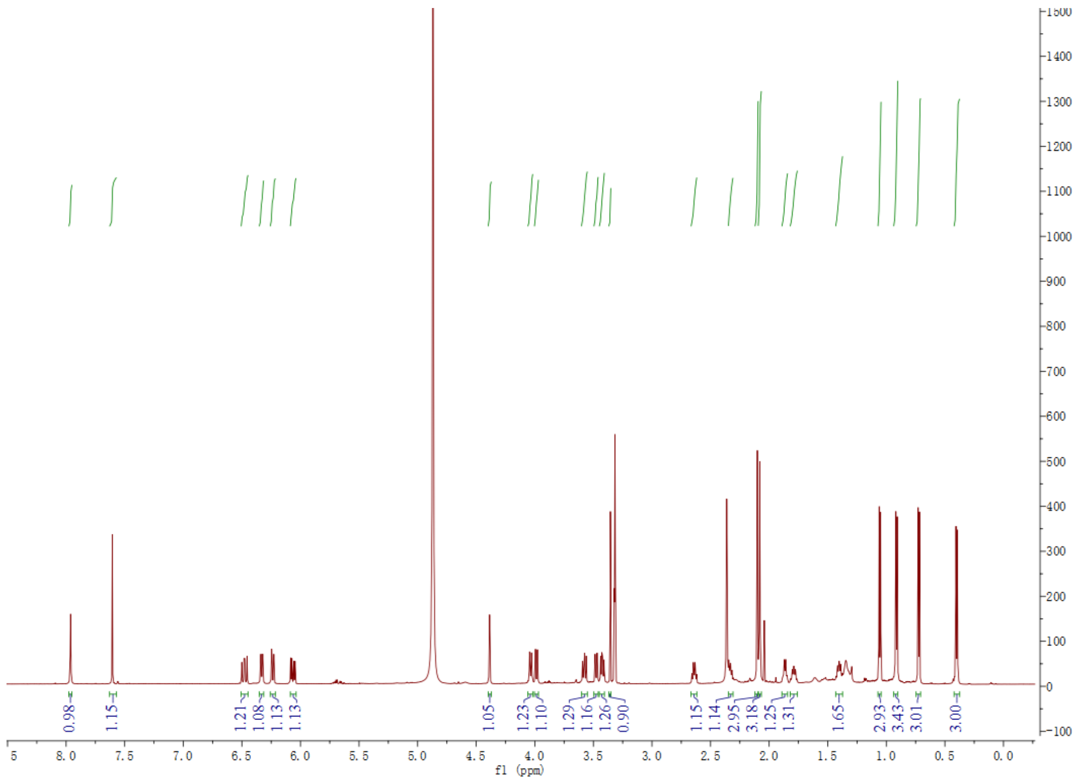

Figure S4. The ^{13}C NMR spectrum of compound **2** in CD_3OD (150 MHz).

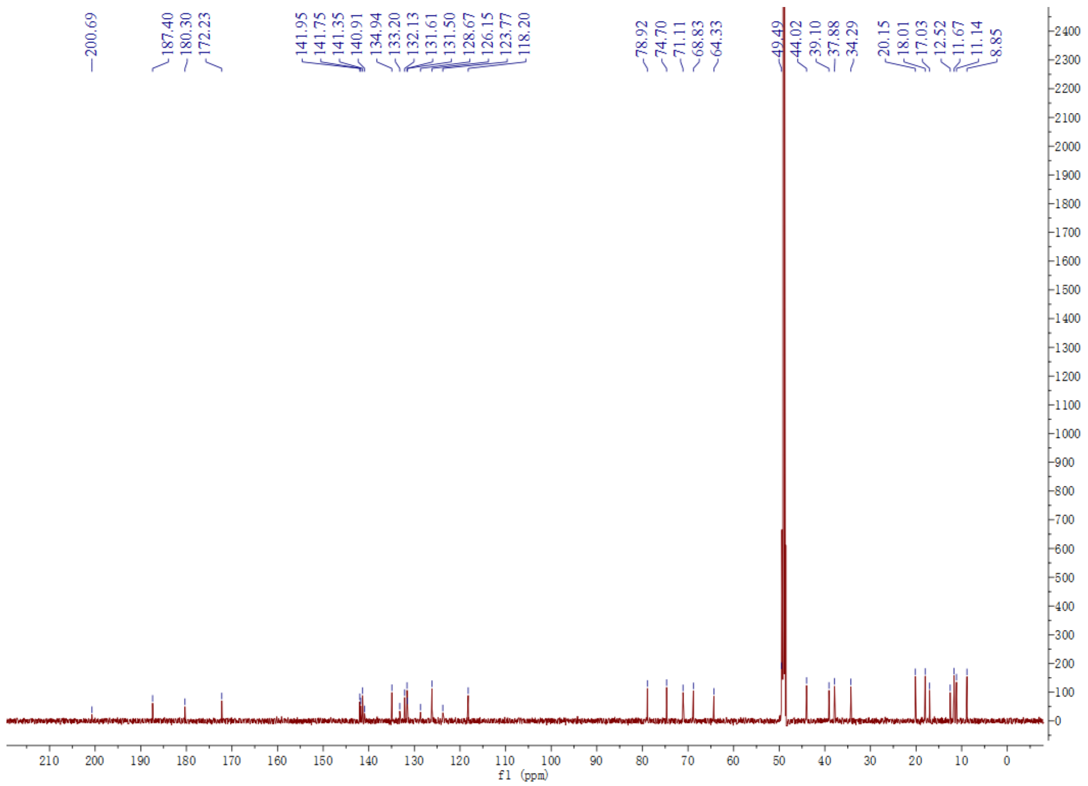

Figure S5. The ^{1}H NMR spectrum of compound **3** in CD_3OD (600 MHz).

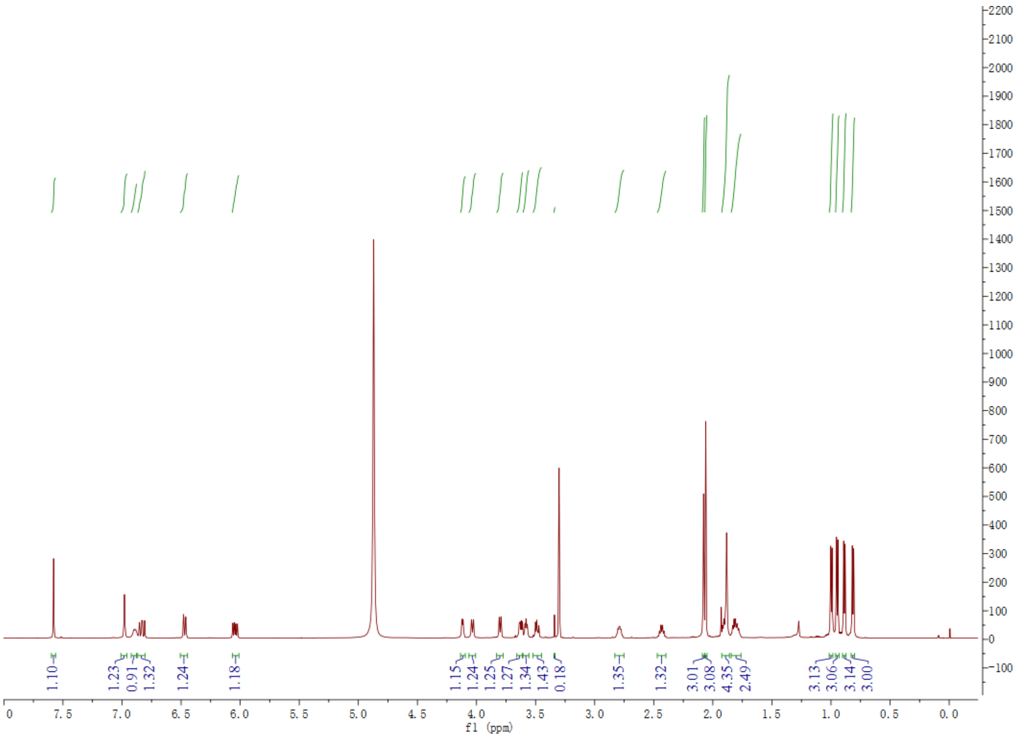

Figure S6. The ^{13}C NMR spectrum of compound **3** in CD_3OD (150 MHz).

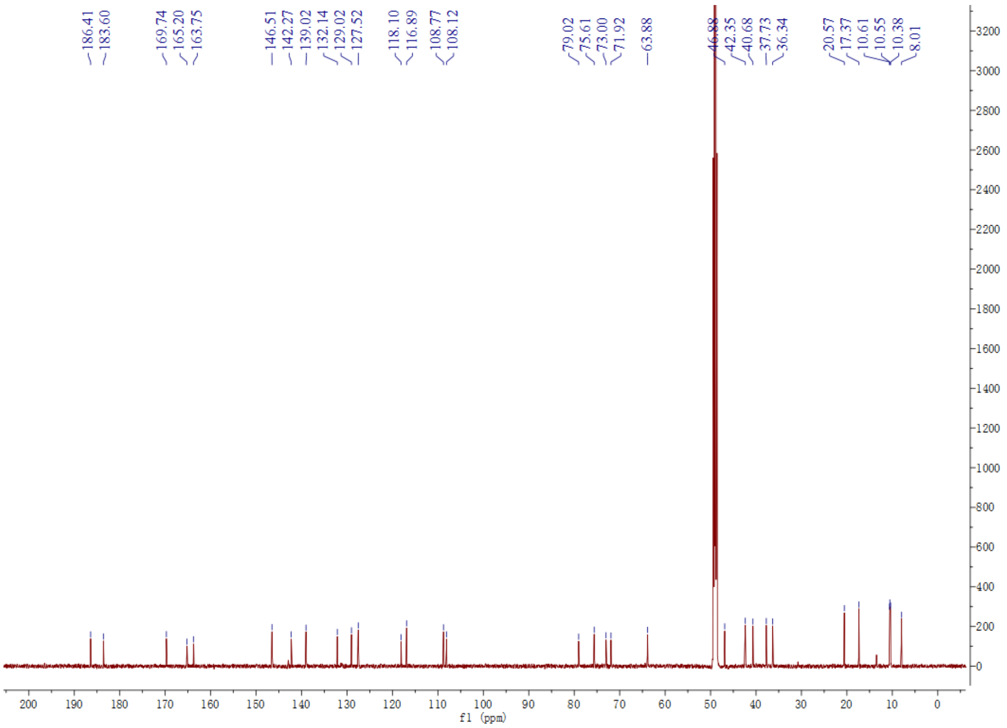

Figure S7. The ^{1}H NMR spectrum of compound **4** in CD_3OD (600 MHz).

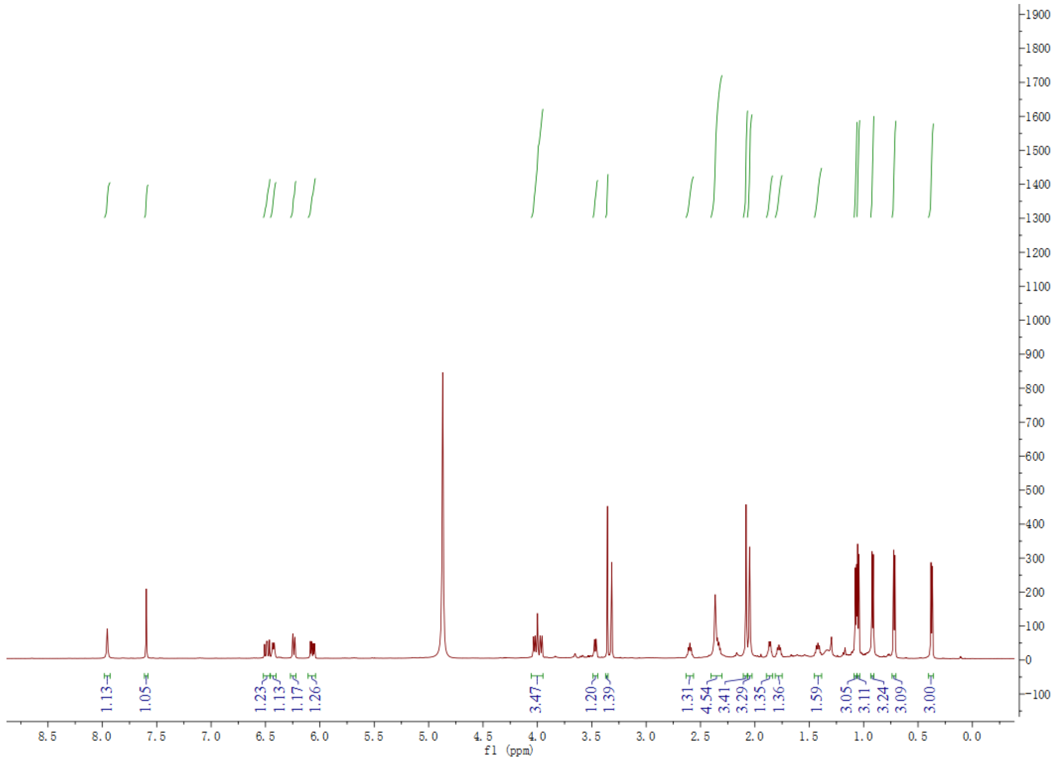

Figure S8. The ^{13}C NMR spectrum of compound **4** in CD_3OD (150 MHz).

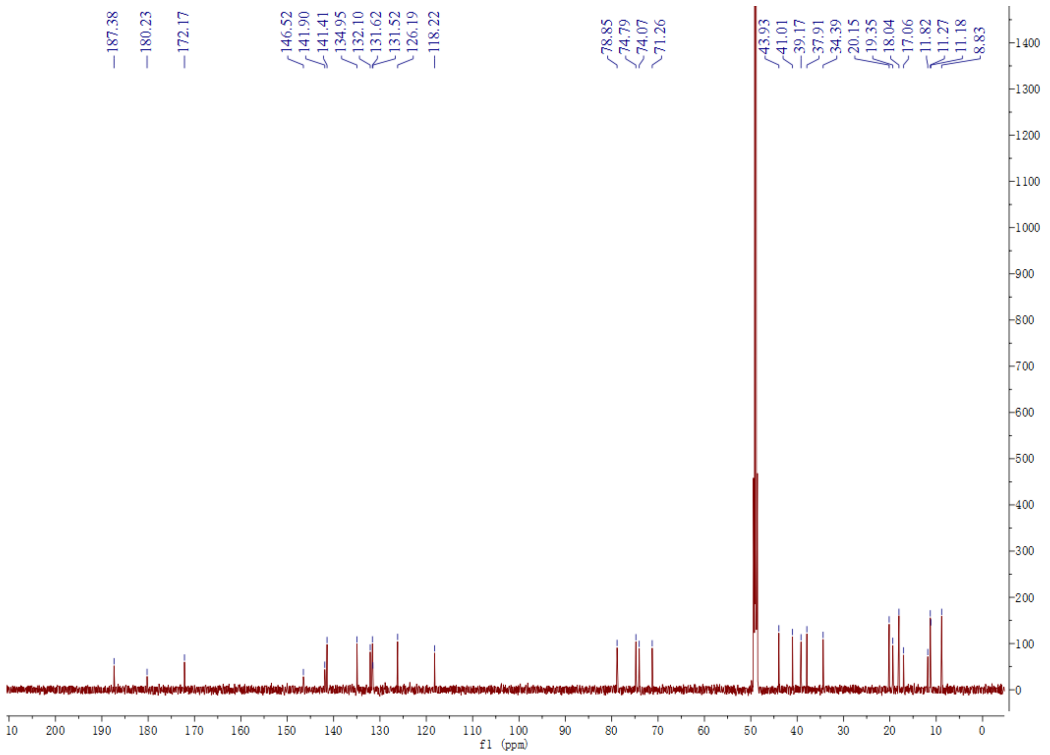

Figure S9. The ^{1}H NMR spectrum of compound **5** in CD_3OD (600 MHz).

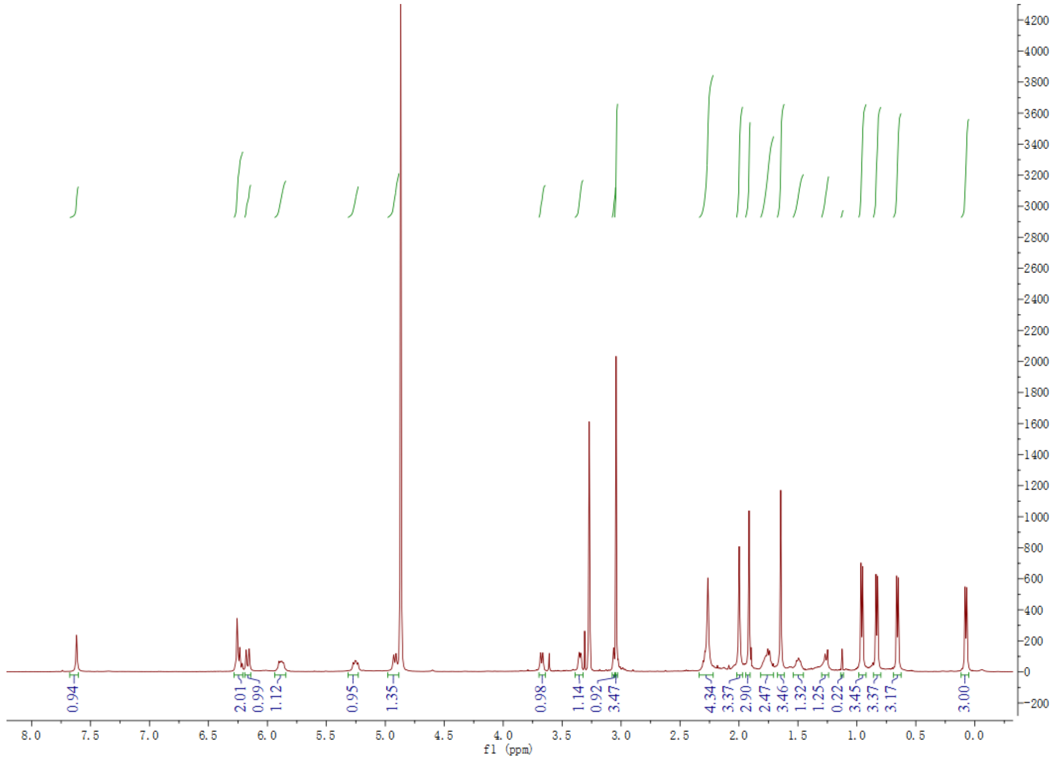

Figure S10. The ^{13}C NMR spectrum of compound **5** in CD_3OD (150 MHz).

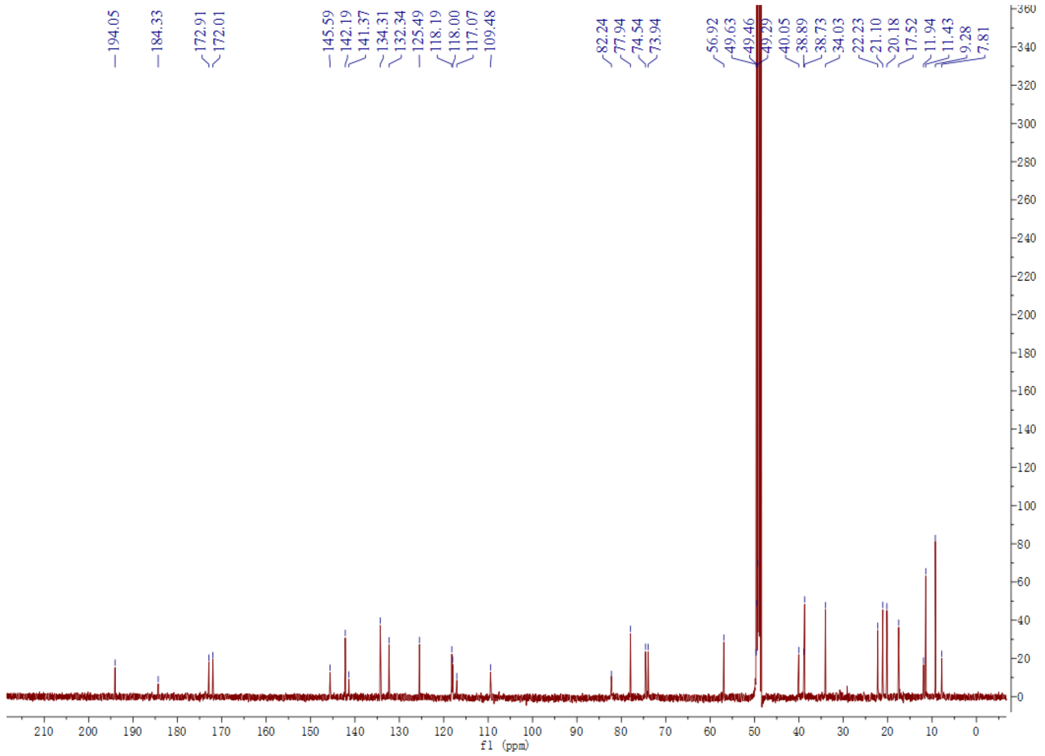

Figure S11. Pictures of representative diffusible pigments produced by actinobacteria in the study.

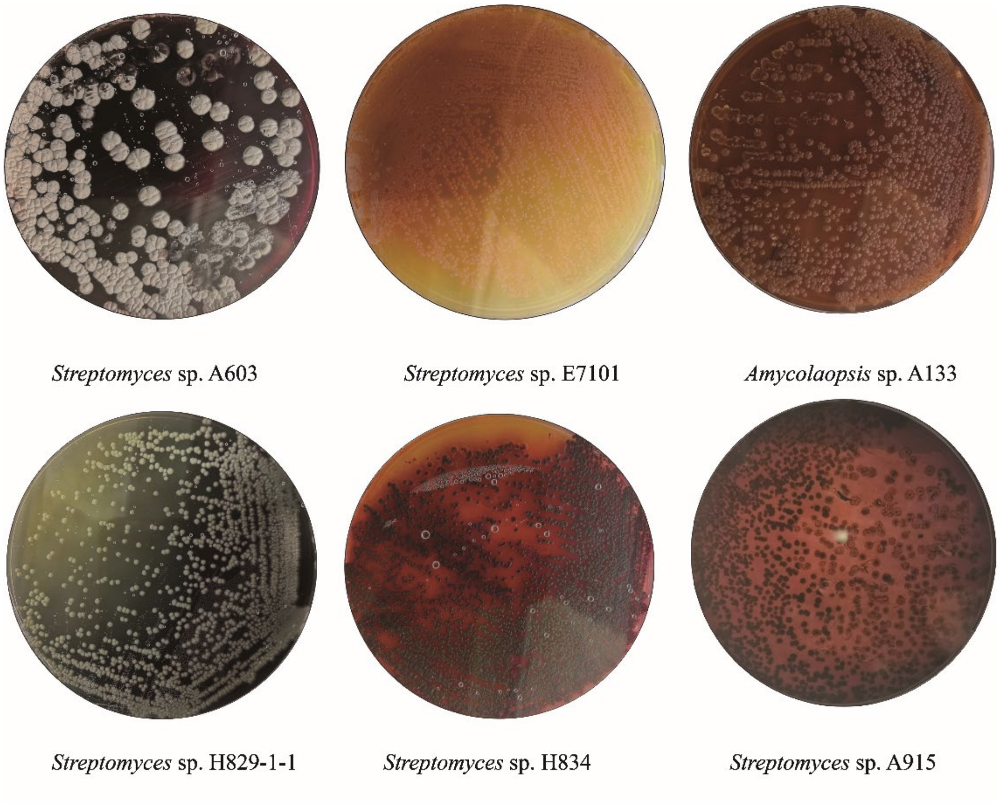

Figure S12. The photo graphs of Amycolatopsis sp. A133 on the plate and picture after Gram-staining. (**A**) The photo graph of *Amycolatopsis* sp. A133 on the plate. (**B**) The picture after Gram-staining of *Amycolatopsis* sp. A133.

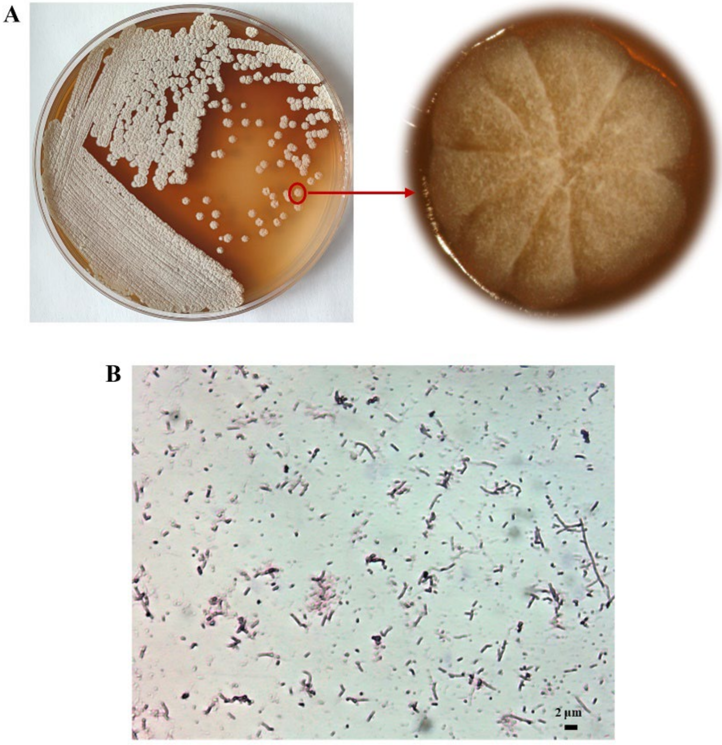

Figure S13. Comparison of the putative biosynthetic gene cluster from strain A133 with the reported rifamycin cluster (BGC0000316).

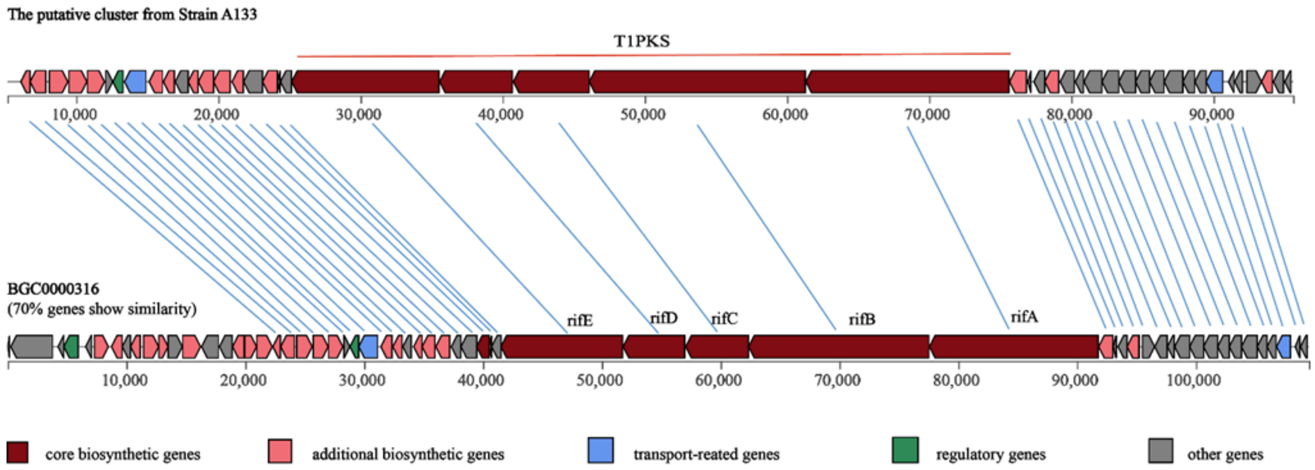

Supplement: Supplementary file 1 [file Data_Sheet_1.pdf]
